# Neurotransmitters and Immunity: Molecular Mechanisms, Biological Functions, Diseases, and Potential Therapeutic Targets

**DOI:** 10.1002/mco2.70556

**Published:** 2025-12-17

**Authors:** Gege Li, Fangfang Li, Yang Tang, Siyu Guo, Yihan Yao, Yuan Fang, Bicheng Zhang, Yu Jiang, Jing Wang, Dang Wu, Jianxia Cheng, Zhihui Huang, Zengfeng Xin, Ting Zhang

**Affiliations:** ^1^ College of Pharmacy Hangzhou Normal University Hangzhou Zhejiang China; ^2^ Cancer Institute (Key Laboratory of Cancer Prevention and Intervention, National Ministry of Education), Second Affiliated Hospital Zhejiang University School of Medicine Hangzhou Zhejiang China; ^3^ Department of Radiation Oncology, Second Affiliated Hospital Zhejiang University School of Medicine Hangzhou Zhejiang China; ^4^ Department of Clinical Laboratory, Second Affiliated Hospital Zhejiang University School of Medicine Hangzhou Zhejiang China; ^5^ Department of Pharmacy Shanghai Sixth People's Hospital Affiliated to Shanghai Jiao Tong University School of Medicine Shanghai China; ^6^ Jiande First People's Hospital Jiande China; ^7^ Department of Orthopedic Surgery, Second Affiliated Hospital Zhejiang University School of Medicine Hangzhou Zhejiang China

**Keywords:** cancer, inflammatory disorders, neurodegenerative diseases, neurotransmitters

## Abstract

Traditionally considered to function solely as signaling molecules within the central nervous system (CNS), neurotransmitters are now recognized as key regulators of systemic homeostasis. They modulate interactions among the nervous, immune, and metabolic systems and influence the development of various diseases. This review systematically summarizes the fundamental properties of major neurotransmitters, including their biosynthesis, receptor subtypes, and key signaling pathways, and analyzes their context‐dependent roles in cancer, neurodegenerative diseases (NDDs), and inflammatory disorders. A primary focus is the three‐dimensional regulatory principle that determines their effects, namely: the receptor type they bind to, cellular microenvironment, and stage of the disease. These factors explain the bidirectional effects of neurotransmitters in disease. This review also evaluates current therapeutic approaches targeting neurotransmitter pathways, ranging from receptor‐specific drugs to emerging combination therapies, and discusses challenges in clinical translation, such as off‐target effects of nonspecific drugs and variable efficacy across disease types. By linking the fundamental mechanisms of neurotransmitter function to clinical challenges, this review provides a comprehensive framework for exploiting the neurotransmitter–immune axis to develop precise therapeutic strategies aimed at improving outcomes in cancer, NDDs, and inflammatory disorders.

## Graphical abstract

Role of neurotransmitters in multiple pathological processes. Various neurotransmitters, including ACh, EPI, NE, 5‐HT, histamine, DA, glutamate, GABA, and neuropeptides participate in regulating key pathological events, including tumor proliferation, tumor migration, remodeling of the immune microenvironment, and the development of NDDs and inflammatory diseases.

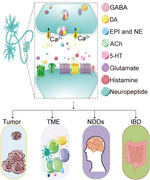

## 1 Introduction

For more than a century, research on neurotransmitters has focused on their roles as chemical messengers in the central nervous system (CNS). After the early 20th century discovery of acetylcholine (ACh) as the first neurotransmitter, studies have consistently linked these molecules to the regulation of core CNS functions, including movement, cognition, and emotion. These studies laid the foundation for understanding neurological disorders such as Parkinson's disease (PD) and depression. This paradigm remained largely unchallenged until the early 2000s, when advances in molecular biology and immunology revealed that neurotransmitters are not exclusive to the CNS; rather, they are also synthesized and secreted in peripheral tissues. For instance, immune cells such as T cells produce ACh [1], intestinal cells synthesize 5‐hydroxytryptamine (5‐HT) [2], and tumor cells release norepinephrine (NE) [3]. These findings redefined neurotransmitters as systemic communication hubs that integrate the nervous, immune, and metabolic systems, a concept termed the neurotransmitter–immune axis, which is central to understanding disease mechanisms beyond the CNS.

Current research has uncovered the diverse roles of major neurotransmitters. Based on chemical composition, neurotransmitters can be classified into four categories: (1) cholines such as ACh; (2) monoamines such as NE, epinephrine (EPI), dopamine (DA), and 5‐HT; (3) amino acids such as glutamic acid and gamma‐aminobutyric acid (GABA); and (4) neuropeptides such as substance P (SP), neuropeptide Y (NPY), and calcitonin gene‐related peptide (CGRP) [4]. Their roles are highly context‐dependent, which contributes to a fragmented understanding of their functions in human pathologies. In the context of cancer, adrenergic stimulation promotes growth in colon, ovarian, and pancreatic cancer (PC); meanwhile, it inhibits melanoma and neuroblastoma, illustrating the complex and tissue‐specific effects of neurotransmitters. In inflammatory disorders, ACh alleviates inflammation in inflammatory bowel disease (IBD) via the cholinergic anti‐inflammatory pathway (CAP) [5], whereas it exacerbates asthma by inducing M3R‐mediated bronchoconstriction [6]. Even within a single disease, neurotransmitters may have stage‐specific effects: α7 nicotinic ACh receptors (α7‐nAChR) reduce β‐amyloid (Aβ) accumulation in early Alzheimer's disease (AD) [7], but worsen tau hyperphosphorylation in later stages [8].

Despite these advances, existing reviews have two critical limitations. They either focus on a single neurotransmitter (e.g., DA in PD) or a single disease (e.g., neurotransmitters in cancer), failing to clarify the neurotransmitter functions across diseases. Furthermore, few reviews link basic biological mechanisms to clinical translational challenges, leaving a gap in guidance for converting preclinical findings into targeted therapies. This gap is particularly significant given the growing interest in neurotransmitter‐targeted interventions and the urgent need for a comprehensive, cross‐disciplinary synthesis.

A key highlight of this review is its integrated perspective and practical orientation. Unlike previous studies that often focused on single dimensions, it systematically outlines the mechanisms of action of various neurotransmitters across diseases and compiles experimental and clinical data on receptor agonists and antagonists. This provides a practical roadmap for developing personalized interventions targeting the neurotransmitter–immune axis, offering a critical reference for refractory cancers, neurodegenerative diseases (NDDs), and inflammatory disorders.

## 2 Neurotransmitters and Diseases

This section adopts the “neurotransmitter–receptor–signaling pathway–disease” relationship as its core framework, systematically analyzing the physiological functions, molecular mechanisms, and pathological roles of key neurotransmitters to provide theoretical support for identifying potential therapeutic targets.

The content is organized with each neurotransmitter treated as an independent analytical unit and follows a unified structure that progresses from physiological foundations to pathological functions. For each neurotransmitter, the discussion first defines its core physiological characteristics, such as biosynthetic pathways, cellular sources, and regulatory networks, to delineate its functional mechanisms under normal physiological conditions. The focus then shifts to three major disease categories (cancer, neurodegenerative disorders, and inflammatory diseases), providing an in‐depth analysis of the neurotransmitter's pathological effects, including the regulation of tumor cell proliferation and metastasis in cancer, modulation of neuronal damage in NDDs, and bidirectional regulation of inflammatory responses in inflammatory disorders. Particular emphasis is placed on two key aspects: the disease specificity of receptor subtypes (for instance, different receptors of the same neurotransmitter may exert either protumor or antitumor effects in cancer) and the crosstalk between signaling pathways.

Specifically, the neurotransmitters discussed include ACh, EPI, NE, 5‐HT, histamine, DA, glutamate, GABA, and neuropeptides. The section underscores their pivotal roles in disease pathogenesis and progression.

### 2.1 ACh

ACh is a cholinergic neurotransmitter of the parasympathetic nervous system that is synthesized and secreted by neuronal or non‐neuronal cells. It is synthesized from choline and acetyl‐CoA by the enzyme choline acetyltransferase (ChAT) [9]. In humans, ACh and ChATs have been identified in epithelial, mesothelial, endothelial, and muscle cells, and ACh synthesis has also been detected in several immune cell types [10]. Functionally, as one of the most essential neurotransmitters in the central cholinergic system, ACh is responsible for maintaining consciousness, learning, and memory. In addition to the brain, peripheral organs are rich in cholinergic innervation, which mediates complex neuroimmune interactions [11]. Non‐neuronal ACh participates in cell proliferation, differentiation, apoptosis, migration, angiogenesis, cytoskeletal function, and immune responses [12]. ACh produced by tumor cells promotes tumor progression through autocrine signaling [11]. Conventionally, ACh exerts muscarinic and nicotinic effects by acting on muscarinic ACh receptors (mAChRs) and nicotinic ACh receptors (nAChRs). Five types of mAChRs (M1R‐M5R) are found in the CNS; these belong to the G‐protein‐coupled receptor (GPCR) superfamily that activates second‐messenger pathways. They can be further classified G‐protein‐coupled type into M1‐like receptors (M1R/M3R/M5R) and M2‐like receptors (M2R/M4R). M1‐like receptors are coupled to Gq/11 proteins, activating the phospholipase C (PLC)/inositol 1,4,5‐trisphosphate (IP3)/diacylglycerol (DAG)/Ca^2+^/protein kinase C (PKC) signaling pathway. M2‐like receptors are coupled to Gi/o proteins, inhibiting the adenylyl cyclase (AC)/cAMP/protein kinase A (PKA) pathway. M1R is mainly expressed in secretory glands (e.g., salivary, mucosal, or sweat glands), M2R in cardiac tissue, and M3R in smooth muscle and secretory glands. mAChRs collectively regulate heart rate, neural transmission, and smooth muscle contraction. nAChRs are Ca^2+^‐ or Na^+^‐permeable ion channels located in muscles and specific autonomic ganglia. They are composed of five homologous transmembrane subunits, represented by distinct α (α2–α10) and β (β2–β4) isoforms [4]. In cancer, M3R and α7‐nAChR are the most frequently reported AChRs [13].

α7‐nAChR functions as an essential Ca^2+^‐dependent channel; Ca^2+^ influx through nAChR promotes intracellular signal transduction and stimulates the release of EPI and NE. Binding of the released EPI and NE to β‐adrenergic receptors (β‐ARs) activates downstream signaling, increasing intracellular cyclic AMP (cAMP) levels and exerting protumor effects [14].

**FIGURE 1:**
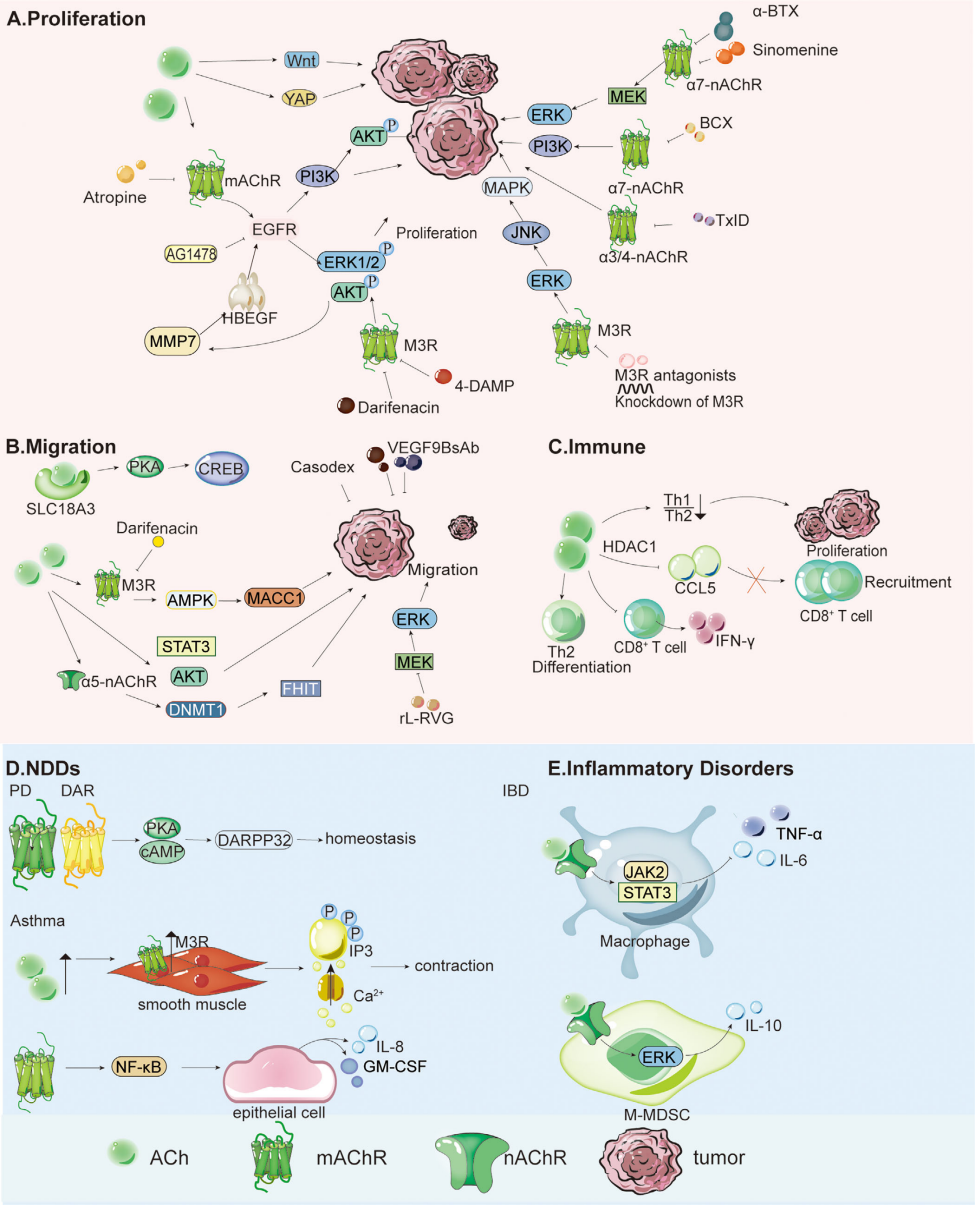
Role and mechanism of ACh in cancer, NDDs, and inflammatory disorders. (A) ACh promotes the proliferation and migration of various tumor cells and exhibits distinct mechanisms in different tumors. In CRC, ACh promotes cell proliferation through the M3R/MMP7/HBEGF/EGFR/ERK1/2 and EGFR/AKT signaling pathways. This effect can be attenuated by AChR antagonists and specific pathway inhibitors. In GC, ACh promotes cell proliferation through activation of the EGFR, Wnt, and YAP signaling pathways. In NSCLC, ACh promotes cell proliferation through the EGFR/PI3K/AKT signaling pathway. (B) In renal cancer, ACh promotes cell migration through the SLC18A3/PKA/CREB signaling pathway. In LUAD, increased ACh levels promote the migration and invasion of LUAD cells by regulating the α5‐nAChR/DNMT1/FHIT axis. In GC, ACh promotes cell migration through the M3R/AMPK/MACC1 signaling pathway. (C) ACh acts on mAChR and nAChR of T cells, DCs, and macrophages through autocrine and paracrine pathways, regulating cytokine production and immune function, thereby indirectly affecting cancer cells. In PDAC, elevated ACh levels promote tumor cell proliferation by generating an immunosuppressive microenvironment with a decreased Th1/Th2 ratio. Mechanistically, ACh disrupts CD8^+^ T cell recruitment via HDAC1‐mediated CCL5 inhibition, directly inhibits CD8^+^ T cell production of IFN‐γ in a dose‐dependent manner, and favors the differentiation of Th2. (D) DA and ACh receptors engage in elaborate crosstalk via the cAMP/PKA/DARPP32 signaling axis to sustain basal ganglia circuit homeostasis. (E) Elevated synaptic ACh levels in patients with asthma enhance binding to M3R on airway smooth muscle, activating the IP3/Ca^2+^ pathway and increasing contraction magnitude. Activated M1R stimulates epithelial cells to secrete inflammatory factors, such as IL‐8 and GM‐CSF, through the NF‐κB pathway, recruiting neutrophils and eosinophils to infiltrate the airways. In IBD, ACh exerts anti‐inflammatory effects via the ENS and CAP. The CAP involves vagal ACh binding to α7‐nAChR on macrophages, inhibiting TNF‐α/IL‐6 via JAK2/STAT3. ACh also activates nAChR/ERK on M‐MDSCs, promoting IL‐10 secretion to exert anti‐inflammatory actions.

Building on the above overview of ACh synthesis, cellular sources, and receptor‐mediated signaling mechanisms, the following sections summarize the roles of ACh in cancer, NDDs, and inflammation, with a focus on its regulatory effects and underlying molecular pathways (Figure 1).

#### 2.1.1 ACh in Cancer

ACh in the tumor microenvironment (TME) promotes tumor proliferation by engaging multiple signaling pathways, including the Wingless‐type MMTV integration site family (Wnt), yes‐associated protein (YAP), mitogen‐activated protein kinase (MAPK), and epidermal growth factor receptor (EGFR)/phosphatidylinositol 3‐kinase (PI3K)/protein kinase B (AKT) pathways in gastric cancer (GC), colorectal cancer (CRC), lung cancer (LC), and non–small‐cell lung cancer (NSCLC; Figure 1A) [15, 16, 17, 18, 19, 20]. In NSCLC, ACh promotes cell proliferation in a dose‐dependent manner through EGFR/P13K/AKT signaling [21]. The activation of Wnt signaling, which is associated with increased proliferation of cancer cells and poor prognosis, was observed in 50% of human NSCLC cell lines and LC excision samples [22]. The α7‐nAChR inhibitor α‐bungarotoxin (α‐BTX) can inhibit NSCLC by suppressing the MEK/ERK signaling pathway [23]. In GC, the proliferation of MGC803 and HGC27 cells was increased by exogenous ACh treatment [20]. Similar to NSCLC, in GC, the EGFR [24], Wnt, and YAP pathways promote tumor progression [25]. mAChR activation can lead to EGFR activation in colon cancer cells even in the absence of EGF. Phosphorylation of extracellular signal‐regulated kinase (ERK)1/2 and AKT induces the formation of intracellular protein complexes that depend on phosphorylated tyrosine, thereby promoting cancer cell proliferation [24]. Among these mechanisms, matrix metalloproteinase 7 (MMP7) catalyzes the release of heparin‐binding EGF‐like growth factor (HBEGF), which mediates ACh‐induced transactivation of the EGFR. This activation promotes colon cancer cell proliferation and downstream ERK signaling, which in turn enhances MMP7 transcription [26]. The specific inhibitor AG1478 blocks the EGFR pathway and inhibits ACh‐induced cell proliferation [15]. In LC, several ACh receptors, such as α3‐nAChR, α4‐nAChR, and α7‐nAChR, are involved. Sinomenine inhibits the expression of α7‐nAChR both in vitro and in vivo, as well as the expression of related signaling molecules (pERK1/2 and ERK1/2) and transcription factors (TTF‐1 and SP‐1), thereby suppressing LC [27]. β‐Cryptoxanthin (BCX) inhibits LC by suppressing the α7‐nAChR/PI3K pathway [28]. Additionally, α‐conotoxin TxID, an inhibitor of α3‐nAChR and α4‐nAChR, also exerts an inhibitory effect on LC (Table 1) [29]. Previous studies have reported that a choline transport inhibitor (hemholinium 3), a nonselective mAChR antagonist (atropine), and a selective M3R antagonist (p‐fluorohexahydrosila‐difenidol hydrochloride) inhibit H508 colon cancer cell proliferation [16]. M3R antagonists, 4‐DAMP and darifenacin, significantly inhibit GC and CRC cell proliferation in vivo [30]. Darifenacin inhibits tumor growth by suppressing ACh‐induced p38, ERK1/2, and AKT signaling [31, 32]. M3R knockdown or antagonism also reduces GC proliferation in nude mouse models, partly by inhibiting ERK/c‐Jun N‐terminal kinase (JNK)‐mediated MAPK pathway activation, highlighting M3R as a promising therapeutic target [33]. This evidence further confirms that M3R in tumors represents a potential target. In summary, ACh is critical in cancer progression, regulating multiple signaling pathways, such as MAPK, Wnt, and YAP, indicating that ACh can be a potential target for cancer treatment.

**TABLE 1:** Current and potential targets cancer treatment through neurotransmitter receptors.

Drug	Type of cancer	Target	Mechanisms of drugs	Refs.
Recombinant Newcastle disease virus (rL‐RVG)	GC	α7‐nAChR	Reducing phosphorylation levels in the MEK/ERK signaling pathway	[34]
α‐Bungarotoxin (α‐BTX)	NSCLC	α7‐nAChR	Inhibiting MEK/ERK	[23]
Sinomenine	LC	α7‐nAChR	Inhibiting pERK1/2, ERK1/2, TTF‐1 and SP‐1	[27]
β‐Cryptoxanthin (BCX)	LC	α7‐nAChR	Inhibiting α7‐nAChR/PI3K	[28]
α‐Conotoxin TxID	LC	α3‐nAChR, α4‐nAChR		[29]
Anti‐α9‐nAChR and methoxy‐polyethylene glycol (mPEG) bispecific antibody (α9 BsAb)	BC	α9‐nAChR	Inhibiting VEGF‐A, p‐VEGFR2, VEGFR2 and MMP9	[35]
Darifenacin	CRC	M3R	Inhibiting p38, ERK1/2, Akt, and MMP‐1	[31]
Darifenacin	GC	M3R		[32]
P‐fluorohexahydrosila‐difenidol hydrochloride	Colon cancer	M3R		[16]
4‐DAMP, darifenacin	GC, CRC	M3R		[30]
Atropine	Colon cancer	mAChR		[16]
Hemholinum 3	Colon cancer	Choline transport		[16]
Quinazoline	PCa	α1‐AR		[36]
Doxazosin and terazosin	PCa	α1‐AR		[37]
Rauwolscine	BC	α2‐AR	Inhibiting p‐ERK1/2	[38]
Salbutamol	BC	β2‐AR	Inhibiting p‐ERK 1/2	[38]
ICI118,551	GC	β2‐AR	Inhibiting β2‐AR/PlexinA1	[39]
L‐748337	Melanoma	β3‐AR	Inhibiting iNOS and NO	[40]
SR59230A	GBM	β3‐AR	Inhibiting SK2/S1P2	[41]
Fenoldopam	BC	D1R	Inhibiting AKT/IGF‐1	[42]
Fenoldopam	BC	D1R	Promoting cGMP/PKG	[43]
SCH23390	LC	D1R	Promoting cAMP/AKT/CREB	[42]
SCH23390	HCC	D1R	Inhibiting cAMP/PI3K/AKT/CREB	[44]
SKF83959	GBM	D1R	Promoting IP3/Ca^2+^	[45]
SKF82958	Ovarian cancer	D1R	Promoting cAMP/PKA	[46]
A77636	BC	D1R	Promoting p‐eIF2α, and inhibiting NFATc1	[47]
Cabergolline	BC	D2R	Inhibiting PI3K/AKT/mTOR	[42]
Domperidone	CRC	D2R	Inhibiting MEK/ERK/STAT3, and inhibiting JAK2/STAT3	[48]
ONC201	GBM	D2R		[49]
ONC201	CRC	D2R		[50]
Haloperidol	PDAC	D2R		[51]
Quinpirole	GC	D2R	Inhibiting IGF‐IR, p‐AKT, increasing KLF4, and inhibiting IGF‐I	[52]
Quinpirole	NSCLC	D2R	Inhibiting ERK1/, AKT, MMP‐9	[53]
Sulpiride	BC	D2R		[54]
Thioridazine	BC	D2R		[55]
Thioridazine	HCC	D2R	Inhibiting EMT	[56]
Thioridazine	Ovarian cancer	D2R		[57]
Trifluoperazine	PCa	D2R	Inhibiting p‐AKT, β‐catenin	[58]
Trifluoperazine	GBM	D2R		[59]
Trifluoperazine	LC	D2R	Inhibiting Wnt/β‐catenin	[60]
Bromocriptine	SCLC	D2R	Inhibiting p‐CREB/p‐STAT4	[42]
Bromocriptine	PCa	D2R	Inhibiting Skp2/c‐Myc	[42]
L‐741,742	GBM	D4R	Inhibiting PDGFRβ, ERK1/2, mTOR	[61]
NAN‐190	PCa	5‐HTR1A		[62]
Pindobind	PCa	5‐HTR1A		[62]
GR127935	GC	5‐HTR1D		[63]
RS127445	CRC	5‐HTR2B		[64]
SB204741	PC	5‐HTR2B		[65]
SB‐269970	PCa	5‐HTR7		[66]
Citalopram	CRC	5‐HT uptake	Inhibiting RhoA/ROCK1/2 and YAP	[67]
Vortioxetine	GC	5‐HTR, serotonin transporter		[68]
Methiotepine, metergoline	SCLC	5‐HTR		[69]
6‐Nitropiperazine, zimeridine, fluoxetine	PCa	5‐HT uptake		[62]
Terfenadine	BC	H1R		[70]
Terfenadine, ranitidine	HCC	H1R, H2R		[71]
Ciproxifan	NSCLC	H3R	Inhibiting PI3K/Akt/mTOR, MEK/ERK	[72]
Clobenpropit	HCC	H3R		[73]
RAMH, ciproxifan	PCa	H3R		[74]
Cimetidine	GC	HR		[75]
Memantine	PCa	NMDAR		[76]
DL‐2‐amino‐5‐phosphonovaleric acid	GC	NMDAR		[77]
MK‐801 (200 mM) or Memantine (80–100 mM)	SCLC	NMDAR		[78]
Memantine (200 mM) and MK‐801	BC	NMDAR		[79]
MK‐801	Melanoma	NMDAR		[80]
Ifenprodil	BC	NMDAR		[81]
MK‐801 (10 mM) or Ifenprodil	GBM	NMDAR		[82]
CPCCOEt	Melanoma	mGluR1		[83]
BAY36‐7620	NSCLC	mGluR1	Inhibiting p‐AKT, bcl‐2, HIF‐1α, VEGF	[84]
Riluzole	BC	mGluR1		[85]
VU0155041	Bladder cancer	mGluR4		[86]
MPEP	GBM	mGluR5	Inhibiting p‐AKT	[87]
Propofol	CRC	GABA_A_ receptor		[88]
Baclofen	HCC	GABA_B_ receptor	Inhibiting cAMP, and promoting p21WAF1	[89]
L‐733,060	PDAC	NK‐1R		[90]
L‐733,060	Melanoma	NK‐1R		[91]
L‐733,060	GC	NK‐1R		[92]
L‐733,060	GBM	NK‐1R		[93]

For metastasis, several studies suggest that ACh in the TME stimulates cancer metastasis in various tumors (Figure 1B). In patients with renal cancer, upregulation of the vesicular ACh transporter solute carrier family 18 member A3 (SLC18A3) enhances ACh accumulation, which activates the PKA/cAMP response element binding protein (CREB) signaling pathway and promotes aggressive cancer cell migration [94]. In GC, ACh drives migration via the M3R/AMP‐activated protein kinase (AMPK)/metastasis‐associated in colon cancer‐1 (MACC1) pathway, which is inhibited by MACC1 knockdown or darifenacin [95]. In addition, recombinant Newcastle disease virus expressing rabies virus glycoprotein (rL‐RVG) inhibits the migration of GC cells and attenuates epithelial–mesenchymal transition (EMT) in vitro via α7‐nAChR signaling. Furthermore, rL‐RVG reduces phosphorylation of the MEK/ERK signaling pathway, downregulates mesenchymal markers N‐cadherin and vimentin, and upregulates the epithelial marker E‐cadherin [34]. In hepatocellular carcinoma (HCC), ACh enhances the migration of HCC cells. ACh upregulates androgen receptors dose dependently to activate signal transducer and activator of transcription 3 (STAT3) and AKT pathways, inhibiting apoptosis; the androgen antagonist Casodex blocks migration [96]. In lung adenocarcinoma (LUAD), elevated ACh levels promote migration and invasion via the α5‐nAChR/DNA methyltransferase 1 (DNMT1)/fragile histidine triad (FHIT) axis [97]. In prostate cancer (PCa), ACh maintains migration through Glyoxalase 1 (Glo1)‐dependent accumulation of 5‐hydro‐5‐methylimidazolone (MG‐H1) and involvement of osteopontin (OPN) [98]. In breast cancer (BC), an anti‐α9‐nAChR/methoxy‐polyethylene glycol (mPEG)‐conjugated bispecific antibody (α9 BsAb) inhibits the expression of VEGF‐A, p‐VEGFR2, VEGFR2, and MMP9, thereby suppressing cell migration [35].

Furthermore, immune cells in the TME, such as lymphocytes, macrophages, mast cells, and dendritic cells (DCs), both produce ACh and express cholinergic receptors, enabling ACh to modulate immunity via autocrine/paracrine signaling (Figure 1C) [1, 99, 100, 101]. In the human tissue lymphoma strain U937, different nAChR subtypes regulate proinflammatory and anti‐inflammatory cytokine production differently. α‐BTX‐sensitive nAChRs (including α7‐nAChR) inhibit tumor necrosis factor α (TNF‐α), while mecamylamine‐sensitive nAChRs suppress *interleukin‐6 (IL‐6)*/*IL‐18*, with both reducing IL‐1β [102]. Therefore, the functions of ACh in the TME are complicated.

Perineural infiltration has been observed in pancreatic ductal adenocarcinoma (PDAC). During severe infiltration, elevated ACh levels promote tumor cell proliferation by producing an immunosuppressive microenvironment with a decreased Th1/Th2 ratio. Mechanistically, ACh disrupts CD8^+^ T cell recruitment through HDAC1‐mediated inhibition of CCL5, directly inhibits CD8^+^ T cell production of IFN‐γ in a dose‐dependent manner, and promotes Th2 differentiation [103].

#### 2.1.2 ACh in NDDs

PD is a multisystem neurodegenerative disorder characterized by substantia nigra (SbN) dopaminergic neuron loss and α‐synuclein (aS) aggregation. Its pathogenesis is rooted in disrupted DA‐cholinergic balance and reduced DA leads to cholinergic overactivity, impairing basal ganglia output and producing motor symptoms such as bradykinesia, rigidity, and tremor (Figure 1D) [104, 105, 106].

mAChR changes in PD are model‐dependent. For instance, Pitx3ak mice lose striatal M2R, while MitoPark mice upregulate M5R [107, 108]. The role of M4R remains controversial. Some studies suggest that reduced M4R worsens symptoms by disrupting ACh release control and D1 receptor (D1R)‐dependent pERK modulation in striatal medium spiny neurons (MSNs) [109]. In contrast, other findings indicate that M4R activation exacerbates symptoms, with antagonists or knockout reducing jaw tremor [110]. nAChRs (α4β2, α7) are consistently reduced (by approximately 50%–90%) in the striatum and SbN, weakening dopaminergic regulation and neuroprotection [107, 111, 112]. Notably, early PD exhibits upregulated cholinergic activity in the striatum and motor supplementary areas, where increased nAChR density acts as a compensatory mechanism to maintain dopaminergic function [113]. This evidence suggests that nAChRs undergo dynamic shifts, early compensation followed by late reduction, throughout PD progression.

Mechanistically, DA and ACh receptors engage in elaborate crosstalk via the cAMP/PKA/DA‐ and cAMP‐regulated phosphoprotein (DARPP32) signaling axis to sustain basal ganglia circuit homeostasis [114, 115]. ACh receptors contribute to this balance through distinct mechanisms: M1‐like receptors trigger Ca^2^⁺ signaling that synergizes with D1‐like receptors to enhance direct pathway medium spiny neuron (dMSN) excitability; M2‐like receptors suppress cAMP production to cooperate with D2‐like receptors in regulating indirect pathway MSNs (iMSNs); and nAChRs indirectly fine‐tune the dMSN/iMSN balance by facilitating DA release [116].

In AD, pathogenesis is strongly linked to cholinergic deficiency. Basal forebrain cholinergic neuron loss reduces ChAT activity and ACh levels, which are closely associated with cognitive decline [117, 118, 119, 120, 121].

For mAChRs, M1R density remains unchanged in AD, but its G‐protein coupling is markedly weakened in the frontal cortex, which correlates with cognitive impairment severity [122]. The mAChR agonist TBBB alleviates AD‐related symptoms in vitro by promoting amyloid precursor protein processing and reducing Aβ production. Furthermore, M3R knockout mice show deficits in conditioned fear learning and memory, confirming the critical role of mAChRs in cognition [123].

For nAChRs, numerous studies reported significantly reduced receptor expression and impaired binding capacity in AD [124, 125]. The marked decrease in α4β2 nAChRs correlates closely with cognitive impairment severity [126]. However, other studies present contrasting results. For instance, Mitsis et al. observed no significant nAChR changes during prodromal and early stages of AD, with reductions occurring only in the late stage [127]. They also noted that α4β2 nAChRs are not directly linked to early cognitive decline in AD [128]. Animal studies further support a complex role for nAChRs in AD. In early‐stage AD models, α7‐nAChRs exert neuroprotective effects by maintaining the septohippocampal cholinergic phenotype, preserving hippocampal integrity, and reducing Aβ accumulation and aggregation [7]. α7‐nAChR knockout mice exhibit significant impairments in odor recognition and attention [129]. Conversely, other studies report that α7‐nAChR knockout mice exhibit neuroprotective effects and improved learning and memory, possibly due to reduced Aβ_1–42_ binding to α7‐nAChRs and decreased α7‐nAChR‐mediated Aβ‐induced tau phosphorylation [8].

AChE promotes Aβ fibril formation. Although AChE inhibitors such as donepezil remain the mainstay of AD therapy, their clinical efficacy is limited [130, 131].

#### 2.1.3 ACh in Myasthenia Gravis

Myasthenia gravis (MG) is an autoimmune disorder causing skeletal muscle weakness via disrupted neuromuscular ACh signaling. Approximately 85% of patients have anti‐nAChR antibodies, which block ACh binding, accelerate receptor internalization/degradation, or activate complement [132, 133, 134].

In addition to anti‐nAChR antibodies, anti–muscle‐specific tyrosine kinase (MuSK) antibodies and anti–low‐density lipoprotein receptor‐related protein 4 (LRP4) antibodies have been identified in some patients with MG [135]. MuSK, a tyrosine kinase receptor located on the postsynaptic membrane, plays a critical role in the aggregation and stabilization of muscle‐type nAChRs. Anti‐MuSK antibodies inhibit MuSK's phosphorylation, thereby blocking the AGRN/LRP4/MuSK/docking protein 7 (DOK7) signaling pathway. This ultimately leads to a marked reduction in muscle‐type nAChRs on the postsynaptic membrane of neuromuscular junctions (NMJs) [136]. As for anti‐LRP4 antibodies, LRP4 acts as a coreceptor for MuSK and is also a key regulator of nAChR aggregation. Anti‐LRP4 antibodies interfere with the interaction between LRP4 and MuSK, exerting an indirect adverse effect on nAChR function [137, 138].

#### 2.1.4 ACh in Inflammatory Diseases

Asthma involves ACh from cholinergic nerves and non‐neuronal cells (epithelia, immune cells), driving airway dysfunction via mAChRs (Figure 1E). Abnormalities include cholinergic fiber hyperplasia, epithelial choline synthase overexpression, and M2 autoreceptor blockade, leading to unregulated ACh release [6, 139, 140, 141, 142, 143]. In terms of specific pathological effects, ACh worsens asthma symptoms via multiple mechanisms:

1. Bronchoconstriction: Elevated synaptic ACh levels in patients with asthma enhance binding to M3R on airway smooth muscle, activate the IP3/Ca^2+^ pathway, and increase contraction magnitude. Concurrently, M2R dysfunction reduces the inhibition of ACh release, resulting in sustained, excessive neurotransmitter release that synergizes with other inflammatory mediators to amplify contractile effects.
2. Mucus secretion: Excessive activation of M1R and M3R is the primary driver. M1R regulates electrolyte and water secretion, whereas M3R directly stimulates submucosal glands to synthesize and secrete mucus. Together, these pathways lead to a marked increase in mucus production in patients with asthma [6, 144, 145].
3. Airway inflammation: Activated M1R stimulates epithelial cells to secrete inflammatory factors such as IL‐8 and granulocyte‐macrophage colony‐stimulating factor (GM‐CSF) through the nuclear factor kappa‐B (NF‐κB) pathway, recruiting neutrophils and eosinophils to infiltrate the airways. Meanwhile, non‐neuronal ACh binds to immune cell receptors, activating these cells and prompting the release of inflammatory mediators, thereby further exacerbating chronic airway inflammation [144, 146].

In IBD, ACh exerts anti‐inflammatory effects via the enteric nervous system (ENS) and CAP (Figure 1E) [147]. As the ENS's primary neurotransmitter, ACh has multiple sources:

(1) The vagus nerve releases ACh via parasympathetic fibers, mainly innervating the intestinal mucosa [148]. (2) Intrinsic ENS cholinergic neurons, accounting for 70% of intestinal neurons, serve as the main local source, directly regulating intestinal immunity [149]. (3) Splenic nerve‐innervated immune cells (e.g., CD4⁺ T cells) also synthesize and secrete ACh, indirectly modulating macrophages via paracrine signaling [150]. Patients with IBD exhibit ENS damage, reduced neurons, decreased ChAT, and low colonic ACh. Activating ChAT⁺ neurons alleviates colitis [5]. The CAP involves vagal ACh binding to α7‐nAChR on macrophages, inhibiting TNF‐α and IL‐6 via Janus kinase 2 (JAK2)/STAT3 and blocking NF‐κB [151, 152, 153, 154, 155]. ACh also activates nAChR/ERK signaling on monocyte‐derived myeloid‐derived suppressor cells (M‐MDSCs), promoting IL‐10 secretion to exert anti‐inflammatory effects [156]. Notably, other nAChR subtypes (beyond α7‐nAChR) also regulate intestinal inflammation. For example, selective α9α10‐nAChR antagonists (e.g., α‐conotoxin RgIA) reduce dextran sulfate sodium (DSS)‐induced colitis severity, possibly by inhibiting immune cell infiltration into inflamed sites [157]. Beyond nAChRs, M3R also influences intestinal inflammation. Under chronic stress, vagal activation via the dorsal motor nucleus (DMV) drives cholinergic enteric neurons to release ACh. ACh then triggers growth arrest and mitochondrial fragmentation via the M3R/MAPK pathway, thereby accelerating the age‐related decline of intestinal stem cells (ISCs) [158]. These findings indicate that distinct AChR subtypes play unique roles in intestinal inflammation, supporting multitarget therapeutic strategies for IBD.

In summary, ACh is a key signaling molecule in the nervous system, immune system, and TME, with disease‐specific and mechanistic functions. In cancer, ACh regulates tumor proliferation and metastasis (e.g., GC, LC) by activating pathways such as MAPK, Wnt, and YAP and synergizing with receptors including α7‐nAChR/M3R. It also shapes an immunosuppressive TME, and related molecules show therapeutic target potential. In NDDs, dysregulated ACh balance is crucial: PD motor symptoms are linked to DA‐ACh imbalance and dynamic nAChR changes (early compensation, late reduction); AD cognitive decline relates to basal forebrain cholinergic neuron loss, abnormal mAChR signaling, and nAChR's dual roles (neuroprotection/pathology). In MG, autoantibodies (anti‐nAChR/anti‐MuSK/anti‐LRP4) block ACh's neuromuscular signaling, causing skeletal muscle weakness. In inflammatory diseases, ACh acts bidirectionally: it exacerbates asthma via mAChR‐mediated bronchoconstriction, mucus hypersecretion, and inflammation, but exerts anti‐inflammatory effects in IBD through the CAP and α7‐nAChR. Overall, ACh/receptor network regulation is central to multiple diseases. Understanding subtype‐specific effects and signal crosstalk will support precise therapeutic strategies.

### 2.2 EPI and NE

EPI and NE are catecholamines derived from the amino acid tyrosine and mediate the fight‐or‐flight stress responses via the sympathoadrenomedullary system. EPI is produced and released by neurons and the adrenal medulla [159], whereas NE is primarily synthesized in the brainstem nuclei by sympathetic nerves and the adrenal gland. NE is mainly released from sympathetic nerve terminals, with a minor contribution from the adrenal medulla [160]. The principal physiological conditions that trigger their release include environmental stimuli, such as temperature fluctuations, and psychological stress [161]. Upon activation, EPI and NE initiate diverse physiological responses, including muscle contraction, glycogen degradation, and airway dilation. NE additionally influences circadian rhythms, dreaming, and learning [162]. The receptors for EPI and NE are classified into two major categories: α‐adrenergic receptors (α‐ARs) and β‐ARs, both of which are GPCRs expressed in most mammalian tissues [163]. α_1_‐ARs are predominantly expressed in smooth muscle and induce Ca^2+^ flux and PKC activation through PLC signaling via Gαq. α_2_‐ARs are present on neurons, smooth muscle, and platelets, functioning as autoreceptors: when activated by NE, they inhibit further NE release through Gαi‐mediated inhibition of cAMP, PKA, exchange protein directly activated by cAMP (EPAC)‐activated guanine exchange protein, and β‐arrestin‐dependent MAPK signaling, processes often implicated in tumor progression and metastasis. There are three subtypes of β‐ARs, each with distinct tissue distributions and functions that govern communication between the extracellular environment and the cell. β_1_‐ARs modulate cardiac output by mediating nerve signals, whereas β_2_‐ARs are expressed in various tissues and exert multiple functions, such as mediating vasodilation in the heart and lung, regulating immune cell transport and effector activity, and contributing to tumorigenesis in epithelial and lymphocytic cells. β_3_‐ARs are mainly expressed in adipose tissue. NE preferentially binds to β_1_‐ARs, while EPI shows higher affinity for β_2_‐ARs [164, 165]. Furthermore, β‐ARs are widely expressed in immune cells, including T and B lymphocytes, NK cells, macrophages, and DCs, where activation generally inhibits immune responses [166, 167], with β_2_‐ARs being the dominant subtype [168].

**FIGURE 2:**
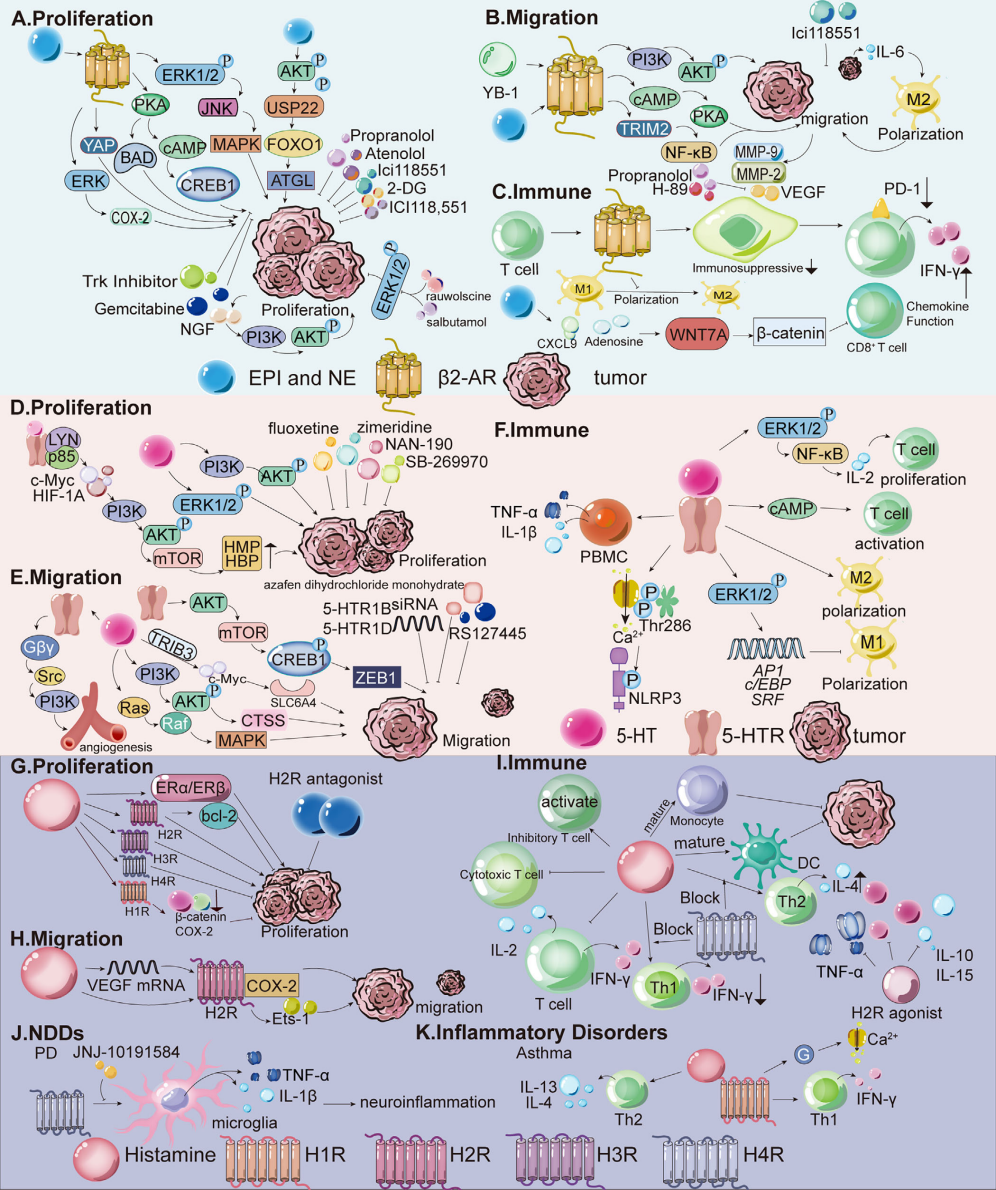
Role and mechanism of EPI, NE, 5‐HT, and histamine in cancer, NDDs, and inflammatory disorders. (A) The effect of EPI and NE on the proliferation differs across tumor types. In GC, EPI and NE promote proliferation through the ERK1/2/JNK/MAPK signaling pathway. In PC, stress hormones such as epinephrine inhibit apoptosis and tumor recurrence through the EPI/β_2_‐AR/PKA/BAD antiapoptotic pathway, thus promoting tumor development. (B) EPI and NE also promote tumor cell migration. In HCC, the transcription factor YB‐1 activates the β_2_‐AR/PI3K/AKT pathway, leading to phosphorylation at serine 102. YB‐1 then translocates to the nucleus, promoting invasion. In ovarian cancer, BC, LC, and colon cancer cells, EPI and NE promote the expression of VEGF, MMP2, and MMP9, thereby enhancing the angiogenesis and migration process of tumor cells. These effects may be attributed to activation of the β‐AR/cAMP/PKA signaling pathway. (C) EPI and NE indirectly modulate T cells by inhibiting recruitment and activation and by polarizing macrophages toward the M2 phenotype. In BC, chronic stress induces T cell and MDSC infiltration in TME, with infiltrated T cells expressing β_2_‐AR on their surfaces. (D) 5‐HT promotes proliferation and migration of tumor cells. In PCa, 5‐HT stimulates proliferation through 5‐HTR1A and 5‐HTR1B and regulates the PI3K/AKT signaling pathway. In PDAC, 5‐HT binding to 5‐HTR2B forms the 5‐HTR2B‐LYN‐P85 complex, activating the PI3K/AKT/mTOR signaling and the Warburg effect by c‐Myc and HIF1A upregulation, increasing enzymes in glycolysis, HMP, and HBP, thereby promoting tumor growth. (E) In NSCLC, 5‐HT promotes metastasis by activating c‐Myc in a TRIB3‐dependent manner. Moreover, c‐Myc 5‐HT transporter SCL6A4 forms a 5‐HT/c‐Myc/SLC6A4/5‐HT feedback loop. In TNBC cells, 5‐HT increases CTSS levels and invasion. 5‐HT also promotes angiogenesis through the 5‐HTR1‐linked Gβγ/Src/PI3K pathway. Knockdown of 5‐HTR1B and 5‐HTR1D via siRNA restrains proliferation, cloning formation, and invasion in PC cells. (F) 5‐HT exerts immunomodulatory effects by polarizing macrophages into an M2‐like phenotype by binding to 5‐HTR2B and 5‐HTR7; 5‐HTR2B inhibits M1 polarization via ERK1/2 phosphorylation, affecting AP1, c/EBP, and SRF transcription. CRC‐derived 5‐HT activates the NLRP3 inflammasome through 5‐HTR3A, inducing Ca^2+^ influx, and CaMKIIα (Thr286) phosphorylation, leading to IL‐1β production and positive feedback on 5‐HT synthesis. 5‐HT and 5‐HTR3 also promote the proliferation and activation of T cells, 5‐HTR1‐mediated cAMP signaling activates T cells; 5‐HTR7 signaling promotes T cell proliferation via the ERK1/2/NF‐κB/IL‐2 pathway. However, 5‐HT can inhibit PBMC TNF‐α and IL‐1β production via LPS‐stimulated 5‐HTR2A. (G) Histamine's effects on tumor proliferation depend on receptor and tumor type. In CRC, GC, and BC, H2R promotes tumor growth, while H4R inhibits it. H3R promotes BC proliferation, whereas H1R inhibits it. In melanoma, H2R promotes proliferation, and H1R inhibits it. (H) Histamine modulates tumor migration. In PC, histamine affects migration via adhesion molecule regulation. In CRC, histamine induces VEGF transcription and activates H2R/COX‐2 signaling, enhancing angiogenesis and migration. In melanoma, H2R activation induces Ets‐1 synthesis, affecting angiogenesis and metastasis. H3R activation promotes BC and HCC metastasis. (I) Histamine also modulates immune responses in tumors. In CRC, it causes Th1 and Th2 imbalance, resulting in increased IL‐4 produced by Th2 and decreased IFN‐γ produced by Th1. This imbalance can be restored by activating H4R in immune cells. In addition, histamine can inhibit the production of cytotoxic T lymphocytes and block the release of IL‐2 and IFN‐γ from T lymphocytes. Histamine may also activate inhibitory T lymphocytes. However, in genetic models such as CRC mice, the use of H2R blockers reduces the production of TNF‐α, IL‐10, IL‐15, and IFN‐γ inflammatory mediators, and the exact mechanism of its immune effect on cancer is still unknown. In BC, the administration of histamine or H4R agonists reduced tumor growth in MDA‐MB‐231 cell‐immunodeficient nude mice. Histamine treatment promotes maturation and intratumoral accumulation of monocytes and DCs, thereby inhibiting the development of mouse lymphoma with EL‐4 cell development. (J) Histamine negatively regulates striatal DA release, possibly via H3R–DAR interactions. H4R is upregulated in PD; its activation stimulates microglia IL‐1β/TNF‐α secretion, worsening neuroinflammation, which can be blocked by H4R antagonist JNJ‐10191584. (K) Histamine affects asthma through receptor‐specific mechanisms. H1R activation triggers G‐protein signaling, Ca^2+^‐dependent smooth muscle contraction (acute bronchospasm), and edema, and regulates Th1 IFN‐γ secretion.

Building on this overview, the following sections summarize the roles of EPI and NE in cancer and NDDs, focusing on their regulatory effects and underlying molecular pathways (Figure 2).

#### 2.2.1 EPI and NE in Cancer

EPI and NE in the TME regulate cancer progression through receptor‐dependent mechanisms that vary by cancer type, influencing proliferation, metastasis, and immune evasion (Figure 2A). Adrenergic stimulation promotes growth in GC, CRC, colon cancer, ovarian cancer, and PC, but inhibits melanoma and neuroblastoma [169]. In GC, HGC27 and MGC803 cells express higher levels of β_2_‐ARs than β_1_‐ARs (β_3_‐ARs are scarce), and EPI/NE stimulate growth via ERK1/2‐JNK‐MAPK signaling. This effect can be blocked by β‐AR antagonists (propranolol, atenolol, and ICI118,551), which inhibit downstream transcription factors (NF‐κB, activator protein 1 [AP‐1], CREB, STAT3) and PlexinA1 [39, 170]. In CRC, EPI drives HT‐29 proliferation through β_1_/β_2_‐ARs/cyclooxygenase‐2 (COX‐2) signaling pathways [171], while in esophageal squamous cell carcinoma, β‐ARs promote progression via ERK/COX‐2 [172]. Chronic stress also activates the β_2_‐AR/PKA/cAMP/CREB1 pathway, inducing transcription of glycolytic enzymes. Stress‐induced cell proliferation and tumor growth can be reversed by treatment with the glycolytic inhibitor 2‐deoxyglucose (2‐DG) and the β_2_‐AR antagonist ICI118,551 [173]. Furthermore, NE promotes the β_2_‐AR‐dependent secretion of nerve growth factor (NGF) by cancer‐associated fibroblasts (CAFs), thereby increasing intratumoral sympathetic innervation and NE accumulation. Adrenergic stimulation also accelerates CRC growth via α_2_‐AR/Gi‐mediated activation of YAP. NGF derived from CAFs directly enhances the growth of CRC cells through the PI3K/AKT pathway. Treatment with tropomyosin receptor kinase (TRK) inhibitors reduces the activation levels of YAP and AKT in mice and attenuates CRC progression [174]. In ovarian cancer, EPI and NE activate β_2_‐AR, regulating cell proliferation and angiogenesis through the β‐AR/cAMP/PKA pathway. The β‐blocker propranolol prevents PDAC development in mice [175]. In PC, EPI inhibits apoptosis via β_2_‐AR/PKA/bcl‐2‐associated death promoter (BAD) signaling, promoting recurrence [164]. In BC, EPI activates AKT signaling, leading to phosphorylation and stabilization of ubiquitin‐specific protease 22 (USP22). By deubiquitinating and stabilizing forkhead box protein O1 (FOXO1), this process promotes adipose triglyceride lipase (ATGL) transcription and expression, thereby accelerating BC progression and metastasis. Inhibition of USP22 combined with β‐blockers exerts synergistic effects in preclinical xenograft BC models [176]. Furthermore, the α_2_‐AR inhibitor rauwolscine and the β_2_‐AR inhibitor salbutamol suppress BC progression by inhibiting p‐ERK1/2 [38]. However, studies have reported variable effects of β‐ARs on BC cell activation [164].

EPI and NE also stimulate the metastasis of tumor cells in BC, PC, and colon cancer (Figure 2B) [177]. In BC, NE induces IL‐6 secretion via a β_2_‐AR‐dependent mechanism, promoting M2 macrophage polarization and enhancing cancer cell migration [178]. In PC, stress‐induced neural activation upregulates invasion‐related genes, facilitating primary growth and dissemination [179]. Both catecholamines downregulate B7‐1/major histocompatibility complex class I (MHC‐I) and upregulate programmed cell death protein 1 (PD‐1)/programmed death ligand 1 (PD‐L1), suppressing antitumor immunity within the TME [180]. In HCC, Y‐box binding protein 1 (YB‐1) activates β_2_‐AR/PI3K/AKT signaling via β‐arrestin 1, triggering YB‐1 nuclear translocation and invasion. YB‐1 disruption mitigates stress‐induced metastasis [181]. In ovarian cancer, BC, LC, and colon cancer, stress‐induced β_2_‐AR activation upregulates vascular endothelial growth factor (VEGF), MMP2, and MMP9 via β‐AR/cAMP/PKA signaling (targeting CREB, NF‐κB, and AP‐1), promoting angiogenesis and migration [175, 182]. The PKA inhibitor H‐89 and propranolol block NE‐induced VEGF, whereas ICI118,551 inhibits metastasis [170, 183, 184]. In CRC, EPI promotes tumor progression and M2 polarization of tumor‐associated macrophages (TAMs) through regulation of the TRIM2‐NF‐κB pathway [185].

EPI and NE also contribute to immune evasion by binding to adrenergic receptors and exerting immunosuppressive effects (Figure 2C). β‐ARs are expressed in most immune cells, including T cells, B cells, NK cells, macrophages, and DCs [166], with β_2_‐AR being the predominant subtype. β_2_‐AR signaling regulates CD8^+^ T‐cell activation and macrophage polarization. Specifically, β‐AR activation inhibits antigen‐specific CD8^+^ T‐cell cytotoxicity and cytokine production [186]. EPI and NE also blunt integrin activation in human CD8^+^ T cells, impeding the recruitment of effector T cells to the TME. In a spontaneous melanoma model, propranolol eliminated T‐cell recruitment [187]. In BC, chronic stress recruits β_2_‐AR⁺ T cells and MDSCs. Propranolol reduces MDSC‐mediated immunosuppression, downregulates PD‐1, upregulates T‐cell‐derived interferon‐γ (IFN‐γ), and enhances anti‐PD‐1 and radiotherapy efficacy [188]. In PC, EPI, and NE, impair T‐cell antigen recognition and induce dysfunction in activated lymphocytes by depleting essential nutrients, such as tryptophan, thereby compromising T‐cell function [189]. Additionally, NE modulates C‐X‐C chemokine ligand 9 (CXCL9) and adenosine secretion via WNT7A/β‐catenin signaling, inhibiting CD8^+^ T‐cell recruitment and activity [190]. Activation of β_2_‐AR signaling also alters the function of TAMs by reducing the deformability of macrophages [191], promoting macrophage infiltration into the TME, inducing M2 polarization [192], and stimulating tumor‐derived chemokine production, which collectively enhances metastasis [193]. Recruited macrophages further stimulate the synthesis of MMPs and VEGF, thereby facilitating stress‐enhanced angiogenesis [194]. In the TME of human BC, elevated β‐AR levels are detected, and NE induces macrophage recruitment to the primary tumor site through α‐AR and β‐AR signaling in a dose‐dependent manner, thereby inhibiting macrophage phagocytosis [195]. As macrophages infiltrate the tumor, NE secretion enhances the metastasis of BC cells to the lymph nodes [192]. Propranolol treatment reverses macrophage infiltration and inhibits tumor dissemination to distant tissues [194].

#### 2.2.2 EPI and NE in NDDs

Central NE neurons are predominantly located in the locus coeruleus, with fibers projecting extensively to the cerebral cortex, hippocampus, and striatum, where they regulate arousal, cognition, emotion, and motor function [196]. The locus coeruleus is affected early by aS pathology in PD, leading to locus coeruleus neuron loss and reduced NE, thereby diminishing noradrenergic projections and exacerbating motor complications during DA replacement therapy [137, 197]. A paradox exists in early PD: although locus coeruleus target regions exhibit fewer noradrenergic fibers, prodromal symptoms such as sleep disturbances and anxiety reflect noradrenergic hyperactivity. This phenomenon likely results from compensatory hyperactivity of damaged locus coeruleus neurons prior to cell death, with subsequent NE deficiency accelerating cognitive decline (causal links remain unconfirmed) [198, 199].

Adrenergic stimulation in PD also has paradoxical effects: inhibition worsens anxiety and depression but may alleviate cognitive impairment, whereas activation reduces anxiety, autonomic dysfunction, depression, pain, and olfactory deficits. These effects correlate with receptor subtype alterations. PD patients show increased α_1_‐ and β_1_‐AR density and reduced α_2_‐AR density [200], with α_2_‐AR activation improving motor function via basal ganglia pathways and enhancing attention and executive function [201, 202].

Similarly, in AD, the locus coeruleus is among the earliest brain regions exhibiting phosphorylated tau accumulation. Experimental locus coeruleus lesions exacerbate neurodegeneration and cognitive deficits in rodent models, and loss of locus coeruleus integrity correlates with human cognitive decline, with the severity of degeneration linked to disease duration [199, 203]. Brain regions receiving locus coeruleus input (hippocampus, frontal, and temporal cortices) exhibit reduced NE levels [204, 205], while EPI levels remain relatively stable [206]. Additionally, terminals positive for tyrosine hydroxylase (TH) and dopamine β‐hydroxylase (DBH) are markedly impaired in AD [207]. However, the simplistic model wherein locus coeruleus damage causes reduced NE transmission is challenged by early AD findings, which indicate that cerebrospinal fluid (CSF) shows elevated NE levels and turnover [199, 208]; this is consistent with prodromal symptoms such as anxiety and sleep disturbances, paralleling PD's “reduced fibers with hyperactive phenotype.” These results suggest complex compensatory mechanisms, as observed in DSP‐4‐induced AD models, where reduced noradrenergic gene expression coexists with increased NE turnover and signaling at terminals [209].

The role of NE depletion in AD remains unclear, as it does not directly drive Aβ accumulation. However, Aβ may induce locus coeruleus degeneration through defective transport of neurotrophins such as brain‐derived neurotrophic factor (BDNF) [210]. NE mitigates Aβ_1–42_‐induced inflammation, and regulates IL‐1β‐mediated amyloid processing [211], and reduced NE may impair microglial Aβ uptake, promoting accumulation in locus coeruleus‐innervated regions such as the hippocampus [212]. NE also limits inflammation and Aβ neurotoxicity, protecting neurons via peroxisome proliferator‐activated receptor delta (PPARδ) activation and glutathione production [213, 214].

Adrenergic receptors are also implicated in AD‐related cognitive decline. Patients exhibit reduced α_2_‐ARs expression in the hippocampal and frontal cortex but increased β_2_‐ARs levels. NE and β_2_‐AR agonists, such as isorepinephrine, promote microglial Aβ uptake and degradation via β_2_‐AR signaling, linking the NE system to AD‐associated neuroinflammation [215].

In summary, EPI and NE are key signaling molecules regulating both the TME and CNS, with their functions mediated via adrenergic receptors and exhibiting disease‐specific mechanisms. In cancer, EPI/NE in the TME predominantly activate pathways such as ERK/MAPK and cAMP/PKA via β_2_‐AR and other receptors, promoting tumor proliferation and metastasis (e.g., GC and CRC) while inhibiting only certain tumors, such as melanoma. They also create an immunosuppressive TME by inhibiting CD8^+^ T cells and inducing M2 macrophage polarization, facilitating immune evasion, which can be reversed by β‐blockers and other agents. In NDDs, both PD and AD involve early damage to NE neurons in the locus coeruleus, exhibiting the paradox of “reduced noradrenergic fibers but compensatory hyperactivity of neurons” in the early stages, contributing to PD motor/cognitive symptoms and AD prodromal discomfort. In PD, α_1_‐ and β_1_‐AR levels increase while α_2_‐AR decreases; in AD, α_2_‐AR decreases and β_2_‐AR increases. NE exerts neuroprotective effects by reducing inflammation and regulating microglial Aβ clearance. Overall, EPI/NE signaling and their receptor networks play a central role in the pathophysiology of both cancer and NDDs. Understanding adrenergic subtype‐specific functions and signaling crosstalk provides a theoretical basis for precise therapeutic interventions.

### 2.3 5‐HT

5‐HT, also known as serotonin, is a monoamine neurotransmitter synthesized from the essential amino acid tryptophan, playing an important role in the CNS and mediating a wide range of physiological activities [216]. It is synthesized by tryptophan hydroxylase (TPH) and dopa decarboxylase (DDC). During 5‐HT synthesis, TPH acts as the rate‐limiting enzyme and exists in two forms: TPH‐1 and TPH‐2. TPH‐1 is primarily expressed in the pineal gland and digestive tract, whereas TPH‐2 is selectively expressed in the brain. DDC is an amino acid decarboxylase with high enzymatic activity in the nervous system and kidneys [217]. 5‐HT regulates multiple CNS functions, including food intake, pain, mood and emotions, sleep, body temperature, sexual behavior, and pituitary endocrine function. Additionally, 5‐HT can induce platelet aggregation in peripheral tissues, trigger immune responses, constrict blood vessels, stimulate smooth muscle contraction, and maintain systemic energy homeostasis [218, 219]. 5‐HT primarily exerts its effects by binding to 5‐HT receptors (5‐HTRs), which are classified into seven subfamilies: 5‐HTR1–7. Each subfamily is further subdivided into subtypes. 5‐HTR1 has five subtypes (A, B, D, E, and F) and is the most prominent member of the 5‐HTR family. Except for 5‐HTR3, a ligand‐gated ion channel, all 5‐HTRs are GPCRs that activate intracellular second‐messenger signaling [220]. 5‐HTRs are expressed across almost all animal species, including humans, and regulate multiple neurotransmitters, thereby influencing diverse biological and neurological processes [221]. Existing studies on the functions of 5‐HT have primarily focused on neurological diseases, including PD, depression, hallucinations, schizophrenia, and anxiety [222].

Building on this overview of 5‐HT, the following sections summarize its roles in cancer, NDDs, and IBD, focusing on its regulatory effects and underlying molecular pathways (Figure 2).

#### 2.3.1 5‐HT in Cancer

5‐HT in the TME can stimulate tumor cell proliferation, as observed in PCa, BC, NSCLC, and small‐cell lung cancer (SCLC; Figure 2D) [223]. In PCa, 5‐HT exerts its effects via 5‐HTR1A, 5‐HTR1B, 5‐HTR2B, and 5‐HTR4, which are expressed in PC3, DU145, and LNCaP cell lines [224, 225, 226, 227]. Notably, 5‐HTR1A and 5‐HTR1B are more abundant in aggressive, high‐Gleason‐grade (4–5) PCa, and 5‐HT drives tumor growth through these two receptors [62, 228]. Silencing DDC or TPH‐1 in 5‐HT‐secreting neuroendocrine cells inhibits proliferation, supporting DDC as a therapeutic target [229]. In BC, 5‐HT inhibits the growth of normal epithelial cells (pHMEC/MCF10A) but exhibits mixed effects on BC cells: high 5‐HT inhibits T47D proliferation, whereas low 5‐HT stimulates MDA‐MB231 proliferation [230]. In SCLC, 5‐HT acts in an autocrine manner as a mitogen, stimulating cell proliferation in a concentration‐dependent manner [231]. 5‐HTR1A and 5‐HTR1D are partially involved in mitotic activity in human SCLC cells [232, 233], likely reflecting complex interactions between the signal transduction pathways of these two 5‐HTRs [234]. Notably, the cumulative effect of their activation by selective agonists accounts for 100% of 5‐HT‐induced mitosis in SCLC cells [233]. In CRC, 5‐HT levels are elevated compared to normal tissues [235]. Proliferation of HT29 cells (a human colon cancer cell line) is concentration‐dependent following 5‐HT incubation. Experimental analyses showed that, compared to normal colon cells, 5‐HTR1A and 5‐HTR1B are overexpressed in CRC tissues but only weakly expressed in HT29 cells [236]. 5‐HTR3 and 5‐HTR4 are expressed in human CRC tumor tissues and HT29 cells [237]. In PCa, 5‐HT regulates the mitotic PI3K/AKT signaling pathway. Studies have reported that 5‐HT increases the phosphorylation of ERK1/2 in a dose‐dependent manner in androgen‐independent cells (PC‐3 and Du145), slows the activation of ERK1/2 in androgen‐dependent LNCaP cells, and induces the phosphorylation of AKT. In DU145 cells, 5‐HT activates the PI3K/AKT signaling pathway [238, 239]. In PC, 5‐HT stimulates PDAC cell proliferation. 5‐HT levels are significantly higher in PDAC tissues than in normal tissues. Mechanistically, 5‐HT binding to 5‐HTR2B stimulates the formation of the 5‐HTR2B‐LYN‐p85 complex, thereby activating PI3K/AKT/mammalian target of rapamycin (mTOR) signaling and promoting the Warburg effect through upregulation of c‐Myc and HIF1A proteins. Subsequently, the levels of metabolic enzymes involved in glycolysis, the pentose phosphate pathway (HMP), and the hexosamine biosynthesis (HBP) pathways are markedly elevated, thereby promoting tumor growth [65, 240]. This mechanism has also been confirmed in GC [241]. Therapies targeting 5‐HTRs include uptake inhibitors (e.g., fluoxetine, zimeridine) and antagonists: NAN‐190 (5‐HTR1A) inhibits DU145/PC3 growth, SB204741 (5‐HTR2B) slows PC progression, and SB‐269970 (5‐HTR7) induces PC‐3 apoptosis [62, 63, 65, 66]. In CRC, 5‐HT enters cells via serotonin transporter (SERT). TG2‐mediated RhoA serotonylation activates Ras homolog gene family member A (RhoA)/Rho‐associated coiled‐coil containing protein kinase 1/2 (ROCK1/2)/YAP signaling, which is inhibited by citalopram [67]. The 5‐HTR2B inhibitor RS127445 also suppresses CRC progression [241].

5‐HT also regulates cancer cell migration (Figure 2E). In NSCLC, 5‐HT may promote cancer cell metastasis. Tu et al. reported elevated 5‐HT levels in metastatic NSCLC sites. Transwell assays and mouse lung metastasis models confirmed that A549 cells exhibit enhanced migration and induce tumor nodules in the lung following 5‐HT stimulation. Additionally, 5‐HT treatment increases colony formation and tumorigenicity in A549 cells. These results indicate that 5‐HT enhances both the tumorigenic and metastatic potential of NSCLC cells. Mechanistically, 5‐HT activates the proto‐oncogene c‐Myc in a tribbles pseudokinase 3 (TRIB3)‐dependent manner. Moreover, c‐Myc upregulates the expression of the SERT SCL6A4, enhancing 5‐HT uptake and forming a 5‐HT/c‐Myc/SLC6A4 feedback loop that amplifies metastatic signaling [242]. In CRC, 5‐HTR2B signaling drives the activation of ribosomal protein S6 kinase B1 (S6K1) via the Akt/mTOR pathway. This activation triggers phosphorylation of CREB1 at Ser133 and its translocation into the nucleus. Phosphorylated CREB1 acts as a transcriptional activator of ZEB1 by binding to the CREB1 half‐site (GTCA) in the ZEB1 promoter, thereby enhancing EMT. As a key regulator of EMT, ZEB1 promotes CRC cell migration and EMT progression. Treatment with the specific HTR2B antagonist RS127445 significantly suppresses lung metastasis and reverses the EMT process [64]. In BC, the expression of cathepsin S (CTSS), a protease involved in tumor angiogenesis, is regulated by the PI3K/AKT and rat sarcoma virus oncogene (Ras)/rapidly accelerated fibrosarcoma (Raf)/MAPK pathways. 5‐HT increases CTSS expression and enhances the invasive capacity of triple‐negative breast cancer (TNBC) cells. Treatment with 5‐HT markedly upregulates CTSS levels and promotes cancer cell invasion. Accordingly, pharmacological inhibition or genetic knockdown of 5‐HT or 5‐HTR signaling significantly suppresses these effects [243, 244]. Moreover, studies have shown that 5‐HT promotes angiogenesis via the 5‐HTR1‐Gβγ/Src/PI3K pathway, although this effect is not inhibited by BJ‐1108, which targets the PI3K/NOX signaling pathway via ERK or p38 [245]. Numerous cancer treatments targeting these pathways have emerged. The uptake inhibitor azafen dihydrochloride monohydrate reduces NSCLC tumorigenicity and inhibits metastasis [242]. Fluoxetine, another 5‐HT uptake inhibitor, has broader effects; it inhibits SERT activity in CRC cells, downregulates lactate transporters, disrupts mitochondrial function, and reduces tumor growth and microvascular density [246]. Additionally, MAPK and PI3K inhibitors, as well as 5‐HTR1A antagonists, can counteract the protumorigenic effects of 5‐HT to varying degrees [239]. Reducing the expression of 5‐HTR1B and 5‐HTR1D in PC cells using siRNA significantly restrains proliferation, cloning formation, and invasion [65, 240].

5‐HT also exerts immunomodulatory effects on cancer cells (Figure 2F). In GC and CRC, 5‐HT acts as a mitogen in the gastrointestinal system, regulating immunity and inflammation by acting on differentially expressed 5‐HTRs in immune cells. This modulation influences cytokine production and release, as well as immune cell activation and proliferation. 5‐HTR2A, 5‐HTR2B, 5‐HTR3A, and 5‐HTR7 are highly expressed in peripheral blood mononuclear cells (PBMC) in GC, and the expression of 5‐HTR3A and 5‐HTR2B is upregulated in tumor tissues compared to adjacent normal tissues. However, a CRC‐specific 5‐HTR subtype has not been explicitly reported [247].

5‐HT polarizes human macrophages toward an M2‐like phenotype via 5‐HTR2B and 5‐HTR7, which are preferentially expressed on anti‐inflammatory M2 macrophages. In macrophages, 5‐HTR2B‐mediated signaling activates ERK1/2, regulating the transcription of macrophage activation‐ and polarization‐related genes such as *AP1*, *c/EBP*, and *SRF*. This signaling inhibits M1 polarization and promotes tumor progression. In addition, CRC cell‐derived 5‐HT enhances activation of the NLRP3 inflammasome in immortalized bone marrow‐derived macrophages (BMDMs) via 5‐HTR3A. Mechanistically, 5‐HT binding to 5‐HTR3A induces Ca^2+^ influx, leading to phosphorylation and activation of CaMKIIα (Thr286), followed by NLRP3 phosphorylation (Ser198) and inflammasome assembly. This activated inflammasome promotes IL‐1β production, which in turn upregulates TPH‐1 transcription in CRC cells, enhancing 5‐HT synthesis and forming a positive feedback loop between 5‐HT and NLRP3 signaling. Silencing TPH‐1 or 5‐HTR3A, or treatment with the TPH‐1 inhibitor 4‐chloro‐L‐phenylalanine or the 5‐HTR3A antagonist tropisetron, alleviates tumor development [248]. Furthermore, 5‐HTR2 inhibitors such as mirtazapine effectively reduce tumor progression by modulating immune mechanisms in the TME. Similarly, 5‐HTR3 inhibitors reduce CRC incidence in mouse models [249].

However, 5‐HT can also impair antitumor immune responses. For example, 5‐HT inhibits TNF‐α and IL‐1β production from LPS‐stimulated PBMCs via 5‐HTR2A, potentially modulating inflammation‐mediated immune responses by regulating 5‐HTR2A expression and IL‐1β production in GC [250]. In another study, 5‐HTR7 activation promoted macrophage polarization toward profibrotic and anti‐inflammatory phenotypes, potentially inhibiting cancer development [251]. 5‐HT also promotes T‐cell proliferation and activation via 5‐HTR3, increasing intracellular Na^+^ levels and facilitating cell cycle progression from the S phase to the G2/M phase. The 5‐HTR3 agonist 2‐methyl‐5HT enhances T‐cell activation through this mechanism [252]. Additionally, 5‐HTR1‐mediated cAMP signaling and [253] 5‐HTR7 activation promotes T‐cell proliferation and activation via the ERK1/2/NF‐κB/IL‐2 pathway [254].

5‐HT plays a multifaceted role in regulating CD8^+^ T‐cell antitumor immunity. CD8^+^ T cells accumulate intracellular 5‐HT for serotonylation through two pathways: de novo synthesis via tryptophan hydroxylase 1 (TPH1) and uptake via the SERT. Tissue transglutaminase 2 (TGM2) catalyzes the transfer of 5‐HT to glutamine residues of glyceraldehyde‐3‐phosphate dehydrogenase (GAPDH), promoting its cytoplasmic localization and inducing a shift toward glycolytic metabolism, thereby enhancing antitumor immunity. Notably, SERT also acts as a negative feedback regulator by inhibiting CD8^+^ T‐cell reactivity through the consumption of 5‐HT secreted by T cells within the TME. Inhibition of SERT using selective serotonin reuptake inhibitors (SSRIs) significantly enhances CD8^+^ T‐cell‐mediated antitumor immunity and reduces tumor growth [255]. Additionally, monoamine oxidase A (MAO‐A), which degrades 5‐HT, serves as an intrinsic negative regulator of CD8^+^ T cells. Adoptive transfer of transgenic T cells (CAR‐T) overexpressing TPH1, which increases 5‐HT production, induces a robust antitumor response [256].

#### 2.3.2 5‐HT in NDDs

Serotonergic neurons originate in the raphe nuclei and project extensively to the basal ganglia, hippocampus, and prefrontal cortex, where they regulate key functions including cognition (spatial navigation, working memory), emotion, sleep, and feeding [257, 258, 259, 260, 261, 262]. In PD, serotonergic neurotransmission declines progressively due to degeneration of the dorsal raphe nucleus, leading to reduced brain levels of 5‐HT and its metabolite 5‐hydroxyindoleacetic acid (5‐HIAA), as well as decreased expression of SERT. Lower SERT levels correlate with longer disease duration [263, 264, 265, 266, 267]. Studies have shown that 5‐HT deficiency in the SbN may contribute to resting tremor, while 5‐HT reuptake inhibition alleviates pain [268, 269].

In addition, receptor‐specific alterations further modulate symptoms. 5‐HTR1A expression is reduced in the basal ganglia, whereas 5‐HTR1B levels remain unchanged [270, 271]. Functionally, 5‐HTR1A/1B receptors promote DA release, while 5‐HT2C receptors inhibit it [272]. Stimulation of serotonergic systems mitigates nonmotor symptoms such as anxiety and constipation via 5‐HTR4‐mediated ACh release, whereas inhibition impairs cognitive function [273]. For motor symptoms, Gi/o‐coupled 5‐HTR1A/1B receptors reduce disturbances by inhibiting glutamatergic neurons and regulating neurotransmitter release [271, 274, 275].

Serotonergic dysfunction also plays a critical role in AD, contributing to behavioral and psychological symptoms of dementia (BPSD) and cognitive decline. AD brains exhibit reduced levels of 5‐HT and its metabolites [276, 277]. In addition, 5‐HTR1A/1B levels decrease with cognitive impairment, while 5‐HTR2A reduction promotes Aβ accumulation, and 5‐HTR4 downregulation in the hippocampus and cortex impairs APP α‐secretase cleavage [278, 279, 280, 281, 282, 283, 284]. Other 5‐HTRs exert protective or modulatory effects: 5‐HTR7 activation reduces neuronal apoptosis [285, 286]; 5‐HTR3 inhibition eases Aβ neurotoxicity [287, 288]; and 5‐HTR6 antagonists improve cognition through reduced Aβ accumulation [289, 290]. 5‐HTR2C has dual effects; its activation reduces Aβ, while inhibition prevents tau phosphorylation [284, 291]. Additionally, binding of 5‐HT to G proteins mediated by TGM2 may influence AD risk by regulating depression, a known precursor to cognitive decline [292].

#### 2.3.3 5‐HT in IBD

Approximately 95% of 5‐HT is produced in the gut, primarily (90%–95%) by enterochromaffin (EC) cells via the TPH1 pathway for local signaling, with the rest synthesized by serotonergic neurons (TPH2‐dependent) that regulate intestinal motility [2].

5‐HT contributes to IBD pathogenesis through immune‐epithelial crosstalk. Proinflammatory factors downregulate SERT (reducing 5‐HT clearance) and increase EC cell numbers (enhancing secretion), forming an “inflammation‐5‐HT accumulation‐inflammation exacerbation” feedback loop. In addition, gut 5‐HTRs (5‐HTR1A, 5‐HTR2A, 5‐HTR3, 5‐HTR4, and 5‐HTR7) regulate inflammatory processes with context‐dependent effects: 5‐HTR1A agonists reduce colitis; 5‐HT2A antagonists alleviate inflammation. The role of 5‐HTR4 remains debated; agonists protect the epithelium, whereas inhibitors improve TNBS‐induced colitis and 5‐HTR7 effects appear dose‐dependent [293, 294, 295, 296, 297, 298, 299]. 5‐HT also modulates immune cells such as lymphocytes and macrophages via 5‐HTRs, promoting IgA^+^ B‐cell differentiation (via 5‐HTR7) and enhancing macrophage phagocytic activity (via 5‐hydroxytryptophan) [300, 301, 302, 303, 304]. Recent studies have shown that gut microbiota interact with the intestinal 5‐HT system. Short‐chain fatty acids (SCFAs) produced by *Bifidobacterium* and *Lactobacillus* upregulate TPH1 expression, and thereby enhance EC‐cell 5‐HT synthesis [305, 306, 307, 308]. Animal models of DSS‐ or DNBS‐induced colitis consistently show elevated gut 5‐HT and reduced SERT, whereas TPH1 inhibition alleviates inflammation [309, 310, 311, 312, 313]. However, clinical findings remain inconsistent, with some studies reporting reduced mucosal 5‐HT in IBD patients, possibly due to disease heterogeneity or sampling bias [314].

In summary, 5‐HT is a key signaling molecule in the TME, CNS, and intestines, acting through diverse 5‐HTRs that exhibit disease‐specific and mechanistically complex functions. In cancer, 5‐HT in the TME activates PI3K/AKT and MAPK pathways via 5‐HTR1A/2B, and other receptors, promoting proliferation and metastasis in PC and CRC. It also bidirectionally shapes the immune microenvironment by polarizing M2 macrophages and regulating CD8^+^ T‐cell function. 5‐HT transporter inhibitors (e.g., fluoxetine) and receptor antagonists (e.g., RS127445) show clear therapeutic potential. In NDDs, PD involves reduced 5‐HT due to degeneration of raphe nucleus neurons, with downregulated 5‐HTR1A/1B linked to motor and nonmotor symptoms. In AD, abnormalities in 5‐HT and its receptors (1A/2A/4) correlate with cognitive decline and Aβ accumulation, while activation of receptors such as 5‐HTR7 exerts neuroprotective effects. In IBD, intestinal 5‐HT (accounting for 95% of the body's total) forms a vicious cycle of inflammation and accumulation, driven by inflammation‐induced SERT downregulation and increased EC‐cell secretion. Overall, the 5‐HTR network lies at the core of pathology across multiple diseases, and subtype‐specific analysis provides a promising basis for precision therapy.

### 2.4 Histamine

Histamine is a widely distributed neurotransmitter and critical biological mediator in mammals that regulates cell invasion, angiogenesis, and immune responses [315]. The amino acid L‐histidine serves as the precursor for histamine synthesis, which requires histidine decarboxylase (HDC), an enzyme expressed by mast cells, EC cells, and neurons [316, 317]. Furthermore, histamine is catabolized by intracellular histamine N‐methyltransferase and extracellular diamine oxidase [318, 319]. Histamine exerts its functions through four receptor subtypes: H1, H2, H3, and H4 receptors (H1R, H2R, H3R, and H4R), each of which belongs to the GPCR family and is differentially expressed or functions in different tissues [320]. H1R is a Gαq/11‐coupled receptor expressed in the gastrointestinal tract, CNS, immune system, cardiovascular system, and genitourinary system. H1R activates Gαq/IP3/Ca^2+^ and Gβγ‐mediated cAMP signaling cascades, thereby regulating processes such as memory, allergic responses, and tumor progression [321, 322]. Similar to H1R, H2R is expressed in almost all peripheral tissues and in the CNS. H2R mediates increased venular permeability, gastric acid secretion, airway mucus production, and inhibition of neutrophil and eosinophil infiltration [323, 324]. Several H1R‐mediated effects, such as smooth muscle contraction, can also be alleviated by H2R [321]. H2R exerts these effects by coupling with AC and activating the cAMP/PKA/CREB signaling cascade. Under GTP‐β‐dependent conditions, H2R can alternatively regulate the phosphoinositol second‐messenger system [325]. H3R and H4R are Gαi/o‐coupled receptors expressed in the CNS and peripheral tissues, whose activation inhibits the cAMP/MAPK signaling pathway [325, 326, 327, 328, 329, 330, 331, 332]. H3R also acts as an autoreceptor and regulates the release of histamine and other neurotransmitters in histaminergic neurons [332].

Based on the above overview, the following sections summarize the roles of histamine in cancer, PD, and asthma, with emphasis on its regulatory effects and underlying molecular pathways (Figure 2).

#### 2.4.1 Histamine in Cancer

Histamine can influence tumor cell proliferation, and its effects vary depending on the receptor subtype and tumor cell type (Figure 2G). In CRC, GC, and BC, H2R exerts a tumor‐promoting effect, whereas H4R inhibits tumor growth; conversely, H2R shows tumor‐inhibiting effects in PC. In OC cells, histamine can promote cell proliferation by upregulating the expression of ERα/ERβ [333]. In CRC, tumors display increased HDC activity, decreased histamine catabolic enzyme activity, and elevated histamine levels compared with normal mucosa [334]. The expression of H1R and H4R is significantly reduced in CRC tissues compared with normal gastrointestinal tissues, whereas H2R levels remain largely unchanged [335, 336, 337]. Among these, H4R may induce cell cycle arrest and consequently inhibit tumor growth and proliferation of CRC tumor cells [336], whereas H2R may promote tumor progression. The role of H1R in CRC remains to be elucidated [334]. Histamine also regulates gastric acid secretion in GC, where gastrin‐regulated HDC expression is markedly elevated [338]. Histamine promotes the proliferation of several gastric tumor cell lines through H2R, and H2R antagonists can induce GC cell apoptosis and inhibit tumor growth in vivo [339]. In contrast, H4R expression is decreased in GC, suggesting a role in tumor progression [328], although its precise function remains unclear. In patients with BC, histamine levels are elevated [340]. Elevated histamine promotes tumor growth via H2R/H3R but inhibits it via H1R/H4R [341, 342]. Similarly, in melanoma cells, histamine promotes cell growth through H2R binding but inhibits cell proliferation through H1R binding [343]. All four histamine receptor subtypes and HDC are expressed in PC (PANC‐I) cells. Studies have shown that the human pancreatic adenocarcinoma cell line PANC‐I overexpresses H1R and H2R [344]. Cricco et al. proposed that H3R is responsible for the proproliferative effects in PC cells, whereas H1R, H2R, and H4R exert the reverse effects. They further suggested that histamine induces G0/G1 phase arrest and regulates Bcl‐2 family proteins via H2R‐mediated antiproliferative activity [345]. In HCC, histamine has dual effects: H1R downregulates β‐catenin/COX‐2 signaling to inhibit growth, H2R promotes proliferation, and H3R's role varies by cell line (antitumor in McA‐RH and protumor in SNU‐368) [71, 73, 315].

The effects of histamine on tumor cell migration are complex (Figure 2H). In PC, histamine negatively regulates cancer cell migration by modulating the expression of different adhesion molecules. This effect was confirmed using H1R and H2R antagonists to enhance PANC‐1 cell adhesion. In addition, increased cell motility was observed in migration assays in the presence of histamine, which may explain the alteration in cell migration [344]. In CRC cells, histamine directly induces VEGF mRNA transcription [346], and activation of the H2R/COX‐2 signaling pathway leads to proangiogenic and promigratory effects [347]. In melanoma cells, histamine promotes the synthesis of E26 transformation‐specific sequence 1 (Ets‐1), a transcription factor, by activating the histamine H2 receptor (H2R). H2R‐mediated upregulation of Ets‐1 enhances angiogenesis and metastasis [343]. In BC and HCC, H3R activation promotes migration via actin cytoskeletal rearrangements, while it reduces cholangiocarcinoma invasion [73].

Histamine can also influence tumor progression by modulating the immune system, with its effects being influenced by the tumor cell type, receptor subtype, and TME (Figure 2I). Evidence indicates that the highest histamine concentrations occur in the stomach, lymph nodes, and thymus. Furthermore, mast cells, basophils, and other types of immune cells have been reported to release intracellular histamine. HDC is also present in these immune cells. These findings indicate that histamine plays a key role in immune regulation [338, 348]. Experimental results have shown that in CRC, two enzymes involved in histamine metabolism are downregulated: diamine oxidase and histamine‐N‐methyltransferase [349]. Exogenous histamine causes an imbalance between Th1 and Th2 lymphocytes in CRC, resulting in increased IL‐4 produced by Th2 and decreased IFN‐γ produced by Th1 [350]. This imbalance can be restored by activating H4R, which is mainly expressed in immune cells and results in antitumor activity [329, 351, 352]. In CRC, histamine can inhibit cytotoxic T lymphocyte production and block the release of IL‐2 and IFN‐γ from T lymphocytes. In addition, histamine may also activate inhibitory T lymphocytes [353]. However, H2R blockers reduce the production of TNF‐α, IL‐10, IL‐15, and IFN‐γ inflammatory mediators in CRC mouse isogenic models, although the precise mechanism underlying these immune effects remains unclear [354]. In BC, histamine/H4R agonists inhibit MDA‐MB‐231 growth in nude mice [355, 356], and histamine promotes monocyte/DC maturation to suppress EL‐4 lymphoma [357].

#### 2.4.2 Histamine in PD

Research on histamine in PD is limited, with effects likely indirect. PD patients exhibit elevated histamine levels in the striatum, substantia nigra reticularis (SNr), and globus pallidus (GP) (Figure 2J) [358]. Histamine negatively regulates striatal DA release, possibly through H3R–DAR interactions [359, 360, 361, 362]. H4R expression is upregulated in PD, and its activation stimulates microglia to secrete IL‐1β and TNF‐α, thereby exacerbating neuroinflammation. This effect can be blocked by the H4R antagonist JNJ‐10191584 [363, 364, 365].

#### 2.4.3 Histamine in Asthma

Histamine is a core inflammatory mediator in asthma, driving bronchospasm, inflammation, and edema via neuroimmune crosstalk. Asthma's immune cascades include allergen‐specific (DC‐induced Th2/IgE/mast cell activation) and nonspecific (epithelial IL‐33/IL‐25 activating group 2 innate lymphoid cells [ILC2s]) pathways (Figure 2K) [366]. Within this neuroimmune network, histamine's central role becomes evident. It drives airway obstruction through three primary mechanisms, inducing airway smooth muscle contraction, accelerating submucosal gland secretion, and causing submucosal edema. These effects are generally achieved through the four histamine receptors (H1R–H4R).

H1R is widely expressed on airway smooth muscle cells, vascular endothelial cells, and immune cells. When bound to histamine, it activates G‐protein signaling, triggering Ca^2+^‐dependent smooth muscle contraction (acute bronchospasm) and edema, and regulating Th1 IFN‐γ secretion [367, 368, 369]. However, H1R antagonists have yet to yield breakthroughs in asthma clinical trials.

H2R is expressed mainly on vascular smooth muscle, glandular cells, and Th2 lymphocytes, and has dual functions. Vascularly, histamine induces vasodilation via H2R, potentially alleviating tissue hypoxia by improving blood flow. Conversely, in vitro studies show that H2R mediates histamine‐induced airway mucin secretion, with excess mucus worsening obstruction. H2R is highly expressed on Th2 cells, and its activation inhibits IL‐4 and IL‐13 secretion, supporting its immune‐regulatory role [369, 370].

H3R's mechanisms remain unclear. Primarily expressed on nerve endings, it indirectly influences airway tone by regulating neurotransmitter release (e.g., ACh), but its role in asthma pathogenesis requires further study. Notably, H3R antagonists do not affect histamine binding to Th1 cells [369].

In contrast, H4R is central to chronic asthma inflammation and highly expressed on eosinophils, mast cells, DCs, and T cells [371, 372]. Their activation enhances eosinophil migration and mast cell recruitment, amplifying immune responses and chronic inflammation [373, 374] while also regulating T cell differentiation and DC function to shape adaptive immunity [375].

In summary, histamine is a key signaling mediator regulating the TME, CNS, and airway inflammation. Its functions are mediated by H1R, H2R, H3R, and H4R, exhibiting disease specificity, receptor dependence, and bidirectionality. In cancer, its effects vary by tumor type and receptor subtype; H2R is mostly protumorigenic (e.g., in CRC and GC) and H4R is mostly tumor‐suppressive (e.g., in CRC), with exceptions in some cases. In PD, histamine levels are elevated in brain regions. H3R negatively regulates DA release, and upregulated H4R promotes neuroinflammation, which can be blocked by antagonists. In asthma, histamine is a core inflammatory mediator: H1R triggers constriction and edema, H2R exerts bidirectional regulation, H4R drives chronic inflammation, and H3R mechanisms remain unclear. Overall, histamine and its receptor network are central to the pathology of multiple diseases. Analyzing receptor subtype‐specific functions may provide a basis for precise therapeutic strategies.

### 2.5 Dopamine

DA is an inhibitory stress neurotransmitter [11] that is converted from tyrosine to L‐DOPA, catalyzed by TH, and then converted to DA by DDC [376]. It is also a precursor in NE and EPI synthesis [11]. In the brain, DA is primarily synthesized by dopaminergic neurons, whereas in the peripheral tissues, it is synthesized and released mainly through the adrenal medulla, mesentery, and sympathetic nerves [376]. DA regulates various physiological functions of the CNS and is involved in multiple pathways such as endocrine regulation, behavioral control, motor control, and cardiovascular function [4]. DA acts as a local chemical messenger in peripheral tissues to regulate blood pressure, kidney function, and pancreatic insulin secretion [377]. Dysregulation of DA‐related signaling pathways may contribute to neurological disorders, such as AD [378], PD [379], migraine [380], multiple sclerosis [381], and schizophrenia [382]. Studies have shown that DA can modulate tumor development [383]. DA exerts its cellular action by binding to DA receptors (DARs), which can be categorized into two groups with different intracellular signaling pathways based on their structure, function, and pharmacology: D1‐like receptors (D1R and D5R) and D2‐like receptors (D2R, D3R, and D4R) [384]. In the nervous system, D1R is reported to be the most highly expressed DAR compared to the other four receptors, while the expression of DARs in other peripheral tissues is still unclear [385]. Signaling involves pathways like AC/cAMP/PKA/CREB, AC/cAMP/PKA/MAPK, and guanylate cyclase/cAMP/PKG: D1‐like receptors increase intracellular cAMP, while D2‐like receptors inhibit it. Beyond cAMP, growing evidence indicates the significant role of additional GPCR signaling cascades in mediating DA's effects. Upon binding to DARs, DA activates the PI3K/AKT signaling pathway, which regulates inflammation‐related cellular responses. Similar to its bidirectional modulation of cAMP signaling, DA can either enhance or inhibit Akt activity, with D2‐like receptors most consistently associated with Akt inhibition. This inhibitory effect is thought to be mediated by β‐arrestin‐dependent recruitment of protein phosphatase 2A (PP2A). Both D1‐like and D2‐like receptors exert comparable influences on IP3‐mediated Ca^2+^ flux. In this pathway, D1‐like receptors couple with Gαq proteins and activate PLCβ/phosphatidylinositol 4,5‐bisphosphate (PIP2)/DAG/IP3/Ca^2+^/PKC/MAPK pathways, ultimately regulating cytokine/chemokine secretion, phagocytosis, and other key cellular functions [43, 377, 386]. These pathways overlap and vary by cell type, contributing to DA's complex effects.

**FIGURE 3:**
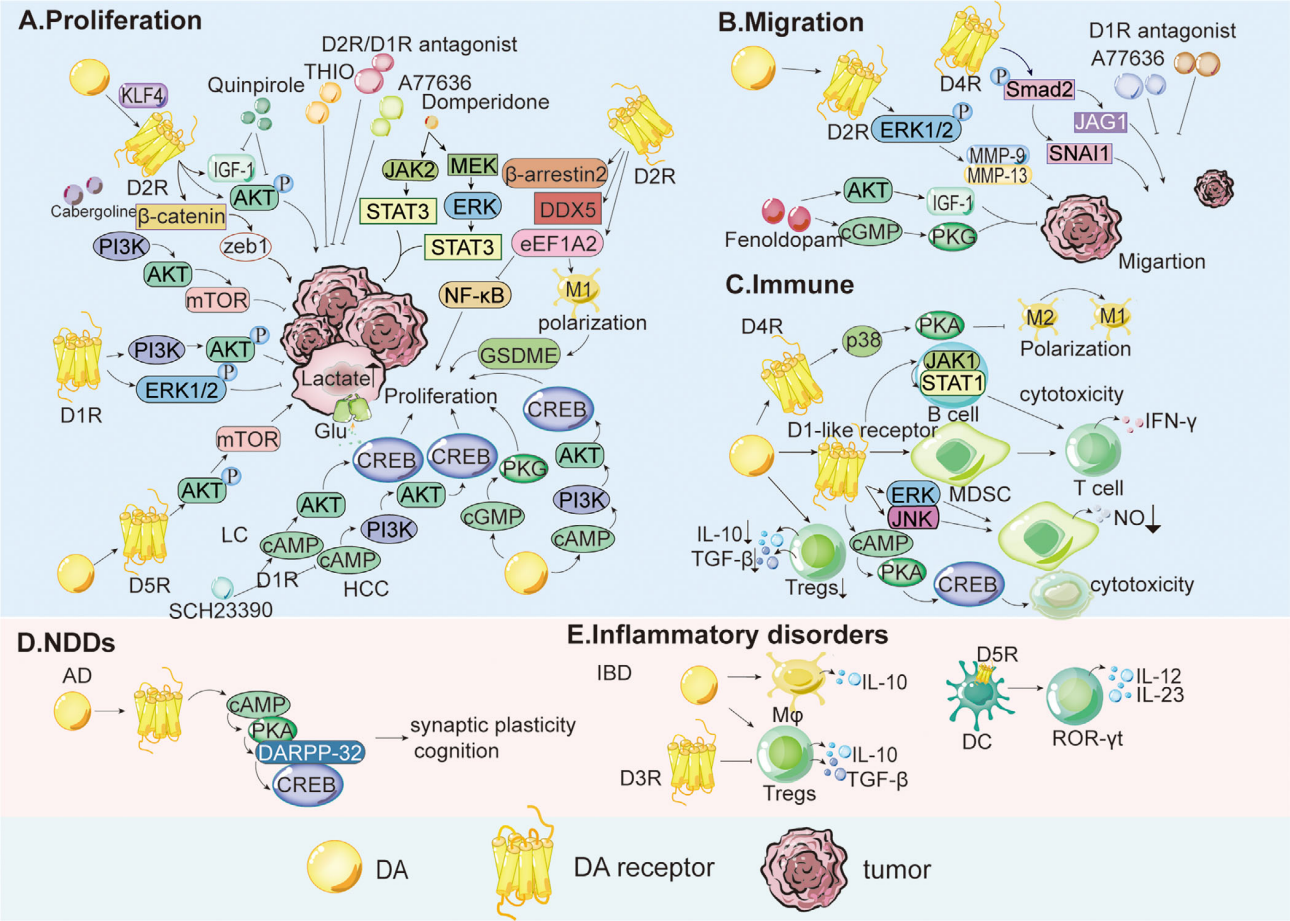
Role and mechanism of DA in cancer, NDDs, and inflammatory disorders. (A) The effect of DA on cancer cell proliferation and migration is tumor‐specific and depends on the DAR subtype. In PC, HCC, and EC, DA promotes tumor cell proliferation, whereas in GC and BC, DA inhibits proliferation. In GC cells, DA upregulates the cell cycle regulator KLF4 through D2R, which downregulates the phosphorylation of the IGF‐1 receptor and AKT, thereby inhibiting IGF‐1‐induced GC cell growth. In LC, SCH23390 promotes the cAMP/AKT/CREB signaling pathway, whereas in HCC, it inhibits the cAMP/PI3K/AKT/CREB signaling pathway. These distinct effects on downstream signaling contribute to inhibition of tumor progression in a context‐dependent manner. (B) The effect of DA on cancer metastasis is tumor‐specific. DA downregulates the ERK‐1/ERK‐2 signaling pathway in endothelial progenitor cells through D2R, reducing MMP‐9 and MMP‐13 expression. D4R interacts with TGFBR1 and TGFBR2, activating the phosphorylation of Smad2 and promoting the nuclear translocation of the Smad2/Smad4 complex. This activates transcription of SNAI1 and JAG1, inducing EMT and enhancing metastatic potential. (C) The effect of DA on immune cells is complex and depends on receptor expression. In the mouse isogenic B16 melanoma model, DA via D1‐like receptors reduces the inhibitory effect of tumor‐induced bone MDSCs on T cell proliferation and IFN‐γ production, enhancing antitumor immunity. Conversely, DA can reduce IFN‐γ‐induced NO production in MDSCs through ERK and JNK signaling by D1‐like receptors. DA also downregulates Tregs, decreases IL‐10 and TGF‐β production, and reduces effector T cell proliferation. In PC, DA inhibits the polarization of tumor‐associated M2 macrophages by inhibiting the PKA/p38 signaling pathway through D4R, thereby improving the efficacy of chemotherapy in vitro and in vivo. (D) In the CNS, reductions in striatal D2‐like receptors and impaired DA reuptake correlate with behavioral abnormalities, whereas D1R and D2R regulate synaptic plasticity and cognition via the cAMP/PKA/DARPP‐32/CREB pathway. (E) In IBD, D3R signaling suppresses Treg immunosuppressive function, reduces anti‐inflammatory IL‐10 production, and promotes Th1/Th17 differentiation. D5R activation in DCs induces ROR‐γt, enhancing IL‐12/IL‐23 secretion and exacerbating Th17‐mediated chronic inflammation. Physiologically, high‐concentration DA activates low‐affinity D1R and D2R to exert anti‐inflammatory effects, including promoting IL‐10 secretion by Tregs and macrophages, thereby enhancing immunosuppression.

Building on the above overview, the following section summarizes the role of DA in cancer, NDDs, and IBD, focusing on its regulatory effects and underlying molecular pathways (Figure 3).

#### 2.5.1 DA in Cancer

The effect of DA on cancer cell proliferation is tumor‐specific and depends on different DARs (Figure 3A) [4]. DA promotes the proliferation of tumor cells in PC, HCC, and esophageal cancer (EC), whereas it inhibits proliferation in GC and BC. Liang et al. reported that D2R expression in PC is abnormally high and that a D2R antagonist can prevent cell proliferation, suggesting that DA promotes the proliferation of PC [51]. Studies have indicated that pimozide (a D2R inhibitor) dose dependently inhibits the growth of PC cell lines, with higher D2R levels corresponding to stronger effects [387]. In EC, DA stimulates cell growth through a D5R‐mediated pathway. DA activation of the DA pathway significantly enhances glucose uptake and lactate production in EC cells. It also promotes extracellular acidification, suggesting that D5R‐mediated activation of the dopsamine pathway triggers the Warburg effect, regulated by the crosstalk between the mTOR and AKT pathways [388]. In GC cells, DA and TH are deficient. Researchers have reported that DA inhibits insulin‐like growth factor 1 (IGF‐1)‐induced GC cell growth by upregulating the cell cycle regulator KLF4 through D2R, in which KLF4 downregulates phosphorylation of the IGF‐1 receptor and AKT. This was corroborated in this study by preconditioning GC cells with quinpirole (a D2R agonist), which inhibited IGF‐1‐induced proliferation in vitro [389]. DA was detected in BC tissues, and D1Rs were elevated in BC cells. Several studies have shown that D1R expression correlates with larger tumors, higher grades, and shorter survival [390]. Interestingly, a study by Clevenger et al. indicates that DA can inhibit BC growth by suppressing prolactin [391]; DA and D1R agonists both restrain the viability of multiple BC cell lines via the cGMP/PKG pathway. This discrepancy may be due to other molecules that activate D1R, which, in turn, activates other pathways that promote cancer development [390]. The D1R agonist A77636 both induces phosphorylated eIF2α (p‐eIF2α) and inhibits nuclear factor of activated T‐cells, cytoplasmic 1 (NFATc1), thereby suppressing BC progression [47]. In addition, high expression of D2R in BC suppresses tumor development by inducing apoptosis and necrosis. Mechanistically, D2R limits NF‐κB signaling activation by interacting with β‐arrestin2, DEAD‐box helicase 5 (DDX5), and eIF2α, and triggers gasdermin E (GSDME)‐mediated pyroptosis by promoting M1 polarization in macrophages [392]. Cabergoline, a D2R agonist, also inhibits the PI3K/AKT/mTOR signaling pathway to suppress BC progression [42]. In human HCC, higher levels of DDC and reduced monoamine oxidase‐A (MAO‐A), a key enzyme for DA degradation, lead to elevated DA levels in MHCC‐97H, SK‐Hep‐1, and MHCC‐97L cell lines. DA activates the cAMP/PI3K/AKT/CREB pathway via D1R in vitro, promoting HCC cell proliferation [393]. The selective D1R antagonist SCH23390 and the DR antagonist thioridazine (THIO) exert antitumor effects in vivo and in vitro [393, 394]. In LC, SCH23390 promotes the cAMP/AKT/CREB signaling pathway [42], whereas in HCC, it inhibits the cAMP/PI3K/AKT/CREB pathway [44]. Notably, these distinct downstream effects both contribute to the inhibition of tumor progression. D1R is expressed in osteosarcoma cells, where it inhibits proliferation by downregulating the ERK1/2 and PI3K/Akt pathways. Overexpression of D1R or treatment with its agonist SKF‐38393 suppresses proliferation [395]. In CRC, D1R–D4R mRNA expression is elevated, with D2R expression highest. D2R knockdown inhibits CRC proliferation via the β‐catenin/ZEB1 signaling pathway, while the D2R antagonist domperidone suppresses tumor growth by blocking the MEK/ERK/STAT3 and JAK2/STAT3 signaling pathways [48]. Conversely, D2R overexpression promotes tumor growth [396]. Activation of D1R and D5R may inhibit tumor growth by inhibiting the AKT/mTOR pathway [397].

D1R plays a significant role in BC cell motility and subsequent metastatic potential. Studies have shown that the selective D1R agonist fenoldopam reduces the migration rate of MDA‐MB‐231 and 4T1 cells, with up to 70% inhibition in MDA‐MB‐231 and BT‐20 cells [398]. Fenoldopam exerts these antimigratory and anti‐invasive effects by inhibiting the AKT/IGF‐1 pathway [42] and activating the cGMP/PKG signaling pathway [43]. A77636, another D1R agonist, also reduces BC cell motility and metastasis, whereas D1R‐specific siRNA eliminates this effect [399]. Sobczuk et al. attributed this to reduced GTPase activity of RhoA and Rac1 and lower levels of EMT markers, MMP‐2, and epithelial cadherin (E‐cadherin) [400].

In addition, DA inhibits tumor metastasis by suppressing angiogenesis. EGF is a key regulator of tumor invasion, migration, and vascular regeneration (Figure 3B). Studies have shown that DA preconditioning prevents EGF‐induced metastasis, partly via D2R [401]. D2R knockout increases tumor angiogenesis [402]. D2R agonists suppress tumor progression in mice by inhibiting angiogenesis and reducing MDSCs [403]. Mechanistically, DA downregulates the levels of MMP‐9 and MMP‐13 by inhibiting the ERK‐1/ERK‐2 signaling pathway in endothelial progenitor cells through D2R [404] and induces endocytosis of VEGFR‐2, impairing VEGF‐A binding, receptor phosphorylation, and downstream signaling [405]. By binding to D2R, DA exerts opposing effects in HCC and PDAC compared with BC. DA promotes migration of PDAC and HCC cells. D2R levels are significantly elevated in PDAC, and knockdown of D2R or inhibition by pimozide or haloperidol induces endoplasmic reticulum (ER) stress and apoptosis while reducing migration [51]. Transwell assays showed that DA promotes HCC cell migration and invasion in vitro [393], while THIO treatment decreases HCC cell motility, likely via reduced expression of MMP3 and MMP9 [394]. In ovarian cancer, DA‐activated D2R, but not D1R, inhibits stress‐mediated tumor growth and microvascular density, whereas D1R activation has no effect [46, 406]. In CRC, D4R interacts with transforming growth factor β receptors (TGFBR1 and TGFBR2), activating the phosphorylation of Smad2 and promoting the nuclear translocation of the Smad2/Smad4 complex, which transcriptionally upregulates SNAI1 and JAG1, inducing EMT and enhancing metastasis [407].

DARs are also expressed in immune cells, including T/B cells, natural killer (NK) cells, monocytes, macrophages, and DCs, enabling DA to regulate immunity via autocrine/paracrine signaling (Figure 3C) [4, 13, 408]. DA activates resting T cells via D1R/D2R/D3R/D5R but inhibits activated T cells via all receptors [409]. It enhances CD8^+^ T‐cell function more strongly than CD4^+^ T cells and boosts antitumor immunity by reducing MDSC‐mediated T‐cell suppression [410]. Increased plasma DA in patients with LC correlates negatively with the proliferation and cytotoxicity of CD4^+^ and CD8^+^ T cells by binding to D1R [411]. Furthermore, plasma DA levels correlate positively with circulating B‐cell counts. Via the DA/D5R/JAK1/STAT1 pathway, DA enhances B‐cell activation and antigen presentation, promoting the expansion of tumor‐specific T cell effectors and cytotoxicity [412].

In the mouse isogenic B16 melanoma model, DA reduced tumor‐induced bone MDSC suppression of T‐cell proliferation and IFN‐γ production through D1‐like receptors, thereby enhancing antitumor immunity. However, further studies have reported that DA also reduced IFN‐γ‐induced NO production in MDSCs by downregulating ERK and JNK signaling via D1‐like receptors [413, 414]. In addition, DA release downregulated Tregs, decreased IL‐10 and transforming growth factor (TGF)‐β production, and reduced Teffs proliferation, without affecting TNF‐α or IFN‐γ production [413, 415]. In PC, DA inhibits M2 macrophage polarization via D4R/PKA/p38, enhancing chemotherapy efficacy [416, 417]. In gliomas, low DA concentrations act through D2R to shift TAM polarization from the M2 phenotype to the M1 phenotype, exerting tumor‐suppressive effects [413, 416]. In BC, the D1‐like receptors/cAMP/PKA/CREB signaling pathway enhances NK‐cell cytotoxicity and inhibits 4T1 tumor growth [418]. In conclusion, DA's effect on immune cells is complex and variable, warranting further investigation.

#### 2.5.2 DA in NDDs

Central dopaminergic neurons are primarily located in the substantia nigra pars compacta (SNc) and ventral tegmental area (VTA). The SNc projects to the striatum via the nigrostriatal pathway, which is critical for motor control, while the VTA innervates the prefrontal cortex and limbic system, regulating emotion and cognition [419]. PD is characterized by SNc dopaminergic neuron degeneration. Approximately 60%–80% neuronal loss leads to striatal DA deficiency and motor symptoms such as rigidity and bradykinesia [420, 421], and the remaining neurons exhibit axonal atrophy and reduced dendritic branching. Nigrostriatal dysfunction impairs activation of the direct pathway and inhibition of the indirect pathway, thereby increasing basal ganglia inhibition of the thalamus and cortex [422]. Nonmotor symptoms, including depression, cognitive impairment, and autonomic dysfunction, result from damage to the mesolimbic and mesocortical pathways [423].

In PD, elevated central aS drives disease progression through two key pathways. First, aS aggregate formation causes SbN dysfunction and dopaminergic neuron loss. The loss of these neurons disrupts the DA regulation and synaptic reuptake via the dopamine transporter (DAT), exposing immune cells to abnormal DA levels that may trigger inflammatory activity [421]. Additionally, excess cytoplasmic DA is degraded by MAO: MAO‐A maintains low cytoplasmic DA levels whereas MAO‐B promotes the formation of reactive oxygen species (ROS) and neurotoxin production, contributing to PD pathogenesis [424]. Increased brain MAO‐B activity in patients with PD accelerates DA oxidation, further damaging dopaminergic neurons. Notably, rasagiline exerts neuroprotective effects in PD models by inhibiting MAO‐B‐mediated ROS formation [425, 426]. Second, aS activates toll‐like receptors (TLRs), initiating microglial and astrocytic activation of NF‐κB and the NOD‐like receptor pyrin domain‐containing protein 3 (NLRP3) inflammasome [427, 428, 429]. The resulting release of inflammatory cytokines (TNF‐α, IL‐6, IL‐1β) and ROS exacerbates aS aggregation and neuronal loss, forming a vicious cycle [430, 431].

The dopaminergic–cholinergic system regulates anxiety, learning, and memory. Patients with AD exhibit significant dopaminergic abnormalities, including reduced DAR density, extracellular DA and its metabolites, and DAT density, indicating neuronal damage (Figure 3D) [206, 432, 433].

Reduction in striatal D2‐like receptors and impaired DA reuptake correlate with behavioral abnormalities. D1R and D2R regulate synaptic plasticity and cognition via the cAMP/PKA/DARPP‐32/CREB pathway [433, 434, 435]. However, findings remain inconsistent; some studies report no D2R changes or increased D3R in the nucleus accumbens. Nonetheless, most evidence links DAR loss with cognitive impairment [436, 437, 438].

Dopaminergic dysfunction contributes to cognitive decline in AD, with D2‐like receptors playing a central role in behavioral regulation. Restoration of dopaminergic tone through subtype‐specific agonists may alleviate apathy and improve cognitive function, although potential off‐target effects on other neurotransmitter systems should be considered.

#### 2.5.3 DA in IBD

DA maintains intestinal homeostasis via the gut–brain axis and neuroimmune interactions, and its dysregulation drives IBD pathogenesis (Figure 3E). Intestinal DA sources include ENS myenteric neurons, epithelial/immune cells, gut bacteria (e.g., lactic acid bacteria), and L‐DOPA conversion [439]. DARs have distinct gut distributions (D4R [mucosa, immune/epithelial cells], D1R/D3R/D5R [ENS endings/mucosa], and D2R [ENS endings]), indicating functional diversity among receptor subtypes [440].

Patients with IBD show disrupted DA metabolism (elevated L‐DOPA, reduced DA, and low DA/L‐DOPA ratios due to diminished L‐amino acid decarboxylase activity), alongside sympathetic nerve fiber loss and impaired DA uptake that worsen deficiency [314, 441]. Animal models confirm D2R agonists reduce inflammation by lowering mucosal permeability [442]. DA also regulates intestinal motility. Specifically, D2R inhibits hypermotility to prevent ulceration [443], and deficiency disrupts motility, exacerbating inflammation [444].

In IBD, reduced DA levels lead to low‐concentration DA preferentially activating high‐affinity D3R, D4R, and D5R, which drives proinflammatory effects [445]. D3R signaling suppresses regulatory T cell (Treg) immunosuppressive function, reduces anti‐inflammatory IL‐10 production, and promotes T helper cell type 1 (Th1)/Th17 differentiation. D5R activation in DCs induces retinoid‐related orphan receptor gamma T (ROR‐γt), boosting IL‐12/IL‐23 secretion and worsening Th17‐mediated chronic inflammation [446]. Under physiological conditions, high‐concentration DA activates low‐affinity D1R and D2R for anti‐inflammatory effects. For instance, D2R activation promotes IL‐10 secretion by Tregs and macrophages to enhance immunosuppression [443]. This concentration–receptor–effect relationship clarifies that reduced DA in IBD disrupts immune homeostasis and drives persistent inflammation.

In summary, DA, a key catecholaminergic signaling molecule regulating the TME, CNS, and intestinal homeostasis, exerts its effects via D1‐like and D2‐like receptors, exhibiting significant disease specificity, receptor subtype dependence, and bidirectionality. In tumors, DA regulation exhibits high heterogeneity. In terms of proliferation, D2R promotes the growth of PC and HCC but inhibits the progression of GC and BC, with controversial roles of D1R in BC. In terms of metastasis, D1R suppresses BC migration, D2R promotes HCC and PC invasion, and D4R induces EMT in CRC. In terms of immune cells, it bidirectionally shapes the microenvironment by regulating the functions of CD8^+^ T cells, MDSCs, and macrophages. Targeted drugs such as SCH23390 and cabergoline have shown intervention potential. In NDDs, dysregulation of the DA system is the core. PD is characterized by the necrosis of dopaminergic neurons in the SNc, with aS aggregation and MAO‐B‐mediated oxidative damage exacerbating DA depletion. AD exhibits downregulated DARs, whose functional loss is associated with cognitive decline, and subtype‐specific agonists may alleviate symptoms. In IBD, intestinal DA metabolic imbalance drives inflammation. Low‐concentration DA activates high‐affinity receptors (D3R/D4R/D5R) to promote inflammation, while physiological‐concentration DA activates low‐affinity receptors (D1R/D2R) for anti‐inflammatory effects. Overall, DA and its receptor network are the pathological core of multiple diseases, and analyzing the functional specificity of subtypes can provide theoretical support for precise treatment.

### 2.6 Glutamate

Glutamate is the primary excitatory neurotransmitter in the CNS, playing essential roles in cognitive function, learning, memory, emotion, sensory processing, and motility [447]. It also participates in neuronal differentiation, migration, and survival of progenitor cells and immature neurons [448]. In non‐neuronal peripheral tissues, glutamate acts as an extracellular signaling molecule to regulate cell growth and development [449]. Glutamate is synthesized from glutamine via aminotransferase or from α‐ketoglutaric acid via glutamate dehydrogenase. It is produced in presynaptic neurons, glia, and even lymphocytes in both the CNS and PNS [450, 451]. Glutamate receptors are classified into two categories: ionotropic glutamate receptors (iGluRs) and metabotropic glutamate receptors (mGluRs). iGluRs are ligand‐gated ion channels permeable to Na^+^, K^+^, and Ca^2+^ upon glutamate binding. Structurally, iGluRs are further subdivided into three groups: N‐methyl‐D‐aspartate (NMDA) receptors, α‐amino‐3‐hydroxy‐5‐methyl‐4‐isoxazolepropionate (AMPA) receptors, and 2‐carboxy‐3‐carboxymethyl‐4‐iso‐propenylpyrrolidine (kainate) receptors. mGluRs are classified into three groups based on sequence homology, pharmacology, and intracellular signaling. Group I mGluR (mGluR1 and mGluR5) is coupled with excitatory Gαq‐like proteins, activating PLC/IP3/DAG/PI3K/AKT or PLC/IP3/DAG/MAPK signaling pathways. However, Groups II (mGluR2 and mGluR3) and III (mGluR4, mGluR6, mGluR7, and mGluR8) negatively couple to AC, reducing cAMP levels [452]. For localization, mGluR7 and mGluR8 are presynaptic, mGluR2 and mGluR3 are at the axon terminals, and mGluR1/mGluR5 are expressed in perisynaptic glia and astrocytes [453, 454, 455]. Glutamate uptake occurs through the glutamate transporters. Excitatory amino acid transporters (EAATs) transport L‐ and D‐aspartate and glutamate from the extracellular space into cells. Conversely, the Na^+^‐independent cystine/glutamate exchanger transports glutamate from the intracellular to the extracellular space while importing cysteine into the cell [456, 457].

**FIGURE 4:**
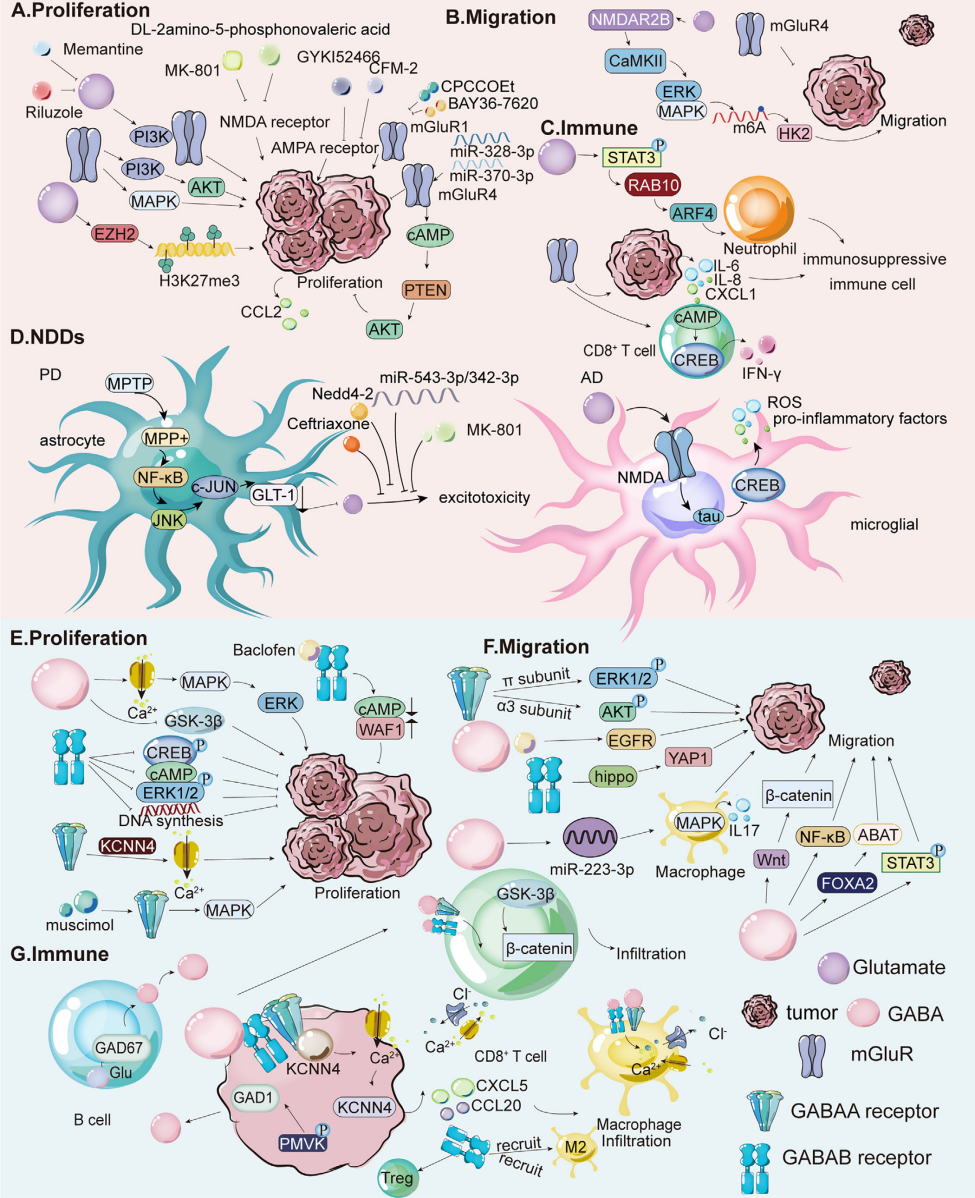
Role and mechanism of glutamate and GABA in cancer, NDDs, and inflammatory disorders. (A) Abnormal glutamate signaling promotes tumor initiation and progression. Glutamate activates MAPK and PI3K/AKT pathways through mGluR, promoting cell proliferation and inhibiting cell apoptosis. However, mGluR4 exerts an antiproliferative effect through the cAMP/PTEN/AKT signaling pathway. (B) Neuronal glutamate can upregulate hexokinase 2 (HK2) expression via mRNA m6A modification through the NMDAR2B/CaMKII/ERK‐MAPK pathway, enhancing glycolysis during tumor metastasis. Expression of mGluR4 is negatively correlated with tumor invasiveness and recurrence. (C) Glutamate suppresses antitumor immunity. It promotes an immunosuppressive neutrophil phenotype via increased pSTAT3/RAB10/ARF4. Activation of mGluR1 induces immunosuppressive chemokines (CXCL1, IL6, and IL8) and activates immunosuppressive immune cells. Inhibition of mGluR4 stimulates CD8^+^ T cells to produce IFN‐γ via cAMP/CREB signaling. (D) Excessive glutamate contributes to DA neuron damage in PD. MPTP is converted to MPP^+^ in astrocytes, activating NF‐κB/JNK/c‐Jun to downregulate GLT‐1, reducing uptake and causing excitotoxicity. Interventions such as ceftriaxone, Nedd4‐2 knockdown, or miR‐543‐3p/342‐3p inhibition mitigate this damage. In AD, elevated glutamate activates extrasynaptic NMDA receptors, upregulates tau, inhibits CREB, and induces microglial ROS/proinflammatory factors, forming an Aβ deposition‐glutamate accumulation‐neuronal injury‐impaired Aβ clearance cycle. (E) The effects of GABA on tumor cell proliferation are cancer‐specific and dependent on the GABA receptor type. The GABA_A_ receptor pathway promotes tumor proliferation, whereas activation of the GABA_B_ receptor pathway inhibits tumor proliferation. Overexpression of GABRP activates the Ca^2+^/MAPK/ERK, stimulating the tumor growth, while GABA_B_ receptor activation strongly inhibits the luciferase activity of p‐CREB and cAMP, isoproterenol‐induced ERK1/2 phosphorylation, and DNA synthesis. (F) GABA_A_ receptor subunits promote migration and invasion: the π subunit activates ERK1/2, and the α3 subunit activates the AKT signaling pathway to promote BC cell migration. However, GABA_B_ receptor activation inhibits tumor cell migration and invasion by blocking Hippo/YAP1 pathways. (G) GABA also modulates antitumor immunity. Interaction between GABA receptors and KCNN4 enhances Ca^2+^ influx, activates KCNN4 signaling, and promotes macrophage infiltration via CXCL5 and CCL20. GABA inhibits DC‐mediated T cell recruitment and GSK‐3β activity, enhancing β‐catenin signaling and reducing CD8^+^ T cell intratumor infiltration. B cells produce GABA upon antigen activation, converting glutamate via GAD67. Secreted GABA binds GABA_A_ receptors on immune cells, opens ion channels, decreases intracellular Ca^2+^ and Cl^−^ concentrations, induces IL‐10(+) macrophages and M2 polarization, and suppresses cytotoxic CD8^+^ T cell activity.

Building on this overview, the subsequent sections summarize the role of glutamate in cancer, NDDs, and IBD, with a focus on its regulatory effects and underlying molecular pathways (Figure 4).

#### 2.6.1 Glutamate in Cancer

As an indispensable amino acid for protein biosynthesis and a precursor for the synthesis of several other amino acids, glutamate serves as the main bioenergy substrate for cell growth (Figure 4A). Owing to uncontrolled proliferation, tumor cells require more energy than normal cells to sustain rapid growth [458]. Previous studies have reported that abnormal glutamate signaling promotes tumor initiation and progression, as cancer cells release excess glutamate that either kills nearby cells or activates glutamate receptors on the tumor cells, causing malignant growth [416, 458]. Glioma cells release neurotoxic glutamate, partly via cystine/glutamate exchangers, to stimulate proliferation; reduced EAAT1/2 (especially EAAT2) in glioblastoma (GBM) impairs glutamate clearance, enhancing accumulation [457, 459, 460]. In BC, tumor‐secreted glutamate upregulates HIF‐1α, contributing to tumorigenesis, and mGluR1 is overexpressed: its knockout or inhibition suppresses proliferation, primarily affecting late‐stage progression [461, 462, 463]. In PCa cell lines (e.g., MATLyLu), glutamate activates mGluR/PI3K signaling to drive growth, whereas riluzole, a glutamate release inhibitor, reduces growth in a dose‐ and time‐dependent manner [464, 465]. Melanoma (B16F1) cells overexpress mGluR1, activating MAPK and PI3K/AKT pathways to promote proliferation and inhibit apoptosis [466, 467]. In HCC, glutamate and its receptor NMDAR2B are significantly elevated, correlating with poor prognosis. Glutamate upregulates CCL2 expression in HCC cells, enhancing TAM recruitment. Mechanistically, this occurs via the NMDAR pathway, which downregulates EZH2 expression, leading to increased CCL2 transcription and subsequent TAM recruitment. Inhibition of the glutamate pathway using MK801 significantly delays tumor growth [468].

Based on the effects of glutamate on tumor proliferation, agonists and antagonists of glutamate have been widely used in clinical research. Glutamate and its receptor antagonists have been reported to inhibit human tumor cell proliferation. Riluzole and memantine inhibit glutamate release and reduce PCa cell growth in a dose‐ and time‐dependent manner [76, 465]. NMDA receptor antagonists, including memantine and MK‐801, reduce cell viability in BC [79], melanoma [80], GBM [82], and drug‐resistant SCLC induced by radiation [78]. Another NMDA receptor antagonist, DL‐2‐amino‐5‐phosphonovaleric acid, inhibits GC progression [77]. NMDA and the AMPA receptor antagonists, such as GYKI52466, exhibit antitumor effects in colon malignant adenoma, astrocytoma, BC, and LC [469]. CFM‐2, an AMPA receptor antagonist, can also inhibit survivin production, thereby suppressing tumor cell growth [470]. Besides iGluRs, mGluR1 antagonists such as CPCCOEt [83] and BAY36‐7620 [84] have demonstrated antitumor effects across various cancer types. Among these, BAY36‐7620 inhibits the expression of p‐AKT, Bcl‐2, HIF‐1α, and VEGF in NSCLC [84]. However, glutamate's role in promoting tumor growth is not absolute. mGluR4 has been identified as a negative regulator [471]. In colon cancer cells treated with 5‐fluorouracil (5‐FU), high mGluR4 expression or agonist treatment promoted cell survival, whereas low mGluR4 expression or antagonist treatment had the opposite effect, indicating resistance to 5‐FU in cells with high mGluR4 levels [472]. Xiao et al. highlighted the antiproliferative role of mGluR4, regulated by miR‐328‐3p and miR‐370‐3p, in BC [473]. Although mGluR4 expression is positively correlated with high‐grade bladder cancer (BCa), functional activation of mGluR4 inhibits proliferation and augments apoptosis through the cAMP/PTEN/AKT signaling pathway, thereby inhibiting BCa development [86].

In PDAC, glutamate levels in tumor tissues are significantly increased, and dysregulation of the mGluR2 subunit of AMPA receptors contributes to a more aggressive and invasive PC phenotype (Figure 4B) [474]. Similarly, mGluR2 dysregulation affects tumor invasion in glioma; the most aggressive gliomas exhibit reduced or absent mGluR2 expression [475]. Clinical studies indicate that mGluR2 expression is lacking in high‐grade malignant GBM, such as ependymoma or medulloblastoma, whereas higher mGluR2 levels are observed in low‐grade astrocytomas [476]. mGluR2‐negative AMPA receptors may be calcium‐permeable; conversely, mGluR2, composed of positively charged amino acids, is impermeable to calcium ions, and intracellular calcium oscillations may trigger adhesive plaque disintegration, promoting glioma cell migration [477, 478]. Furthermore, neuron‐released glutamate can upregulate hexokinase 2 (HK2) expression via mRNA m6A modification through the NMDAR2B/CaMKII/ERK‐MAPK pathway, enhancing glycolysis in PDAC cells and promoting perineural migration (PNI). Dual‐targeted nanoparticles developed by the same group, targeting mGluR2 and NMDAR pathways, effectively block PNI in PDAC [479, 480]. PCa serum glutamate correlates with Gleason score [481]. In LC, activation of mGluR1 signaling promotes brain metastasis. This effect is mediated by LC–astrocyte interactions, inducing high expression of mGluR1 in LC cells via the Wnt‐5a/prickle planar cell polarity protein 1 (PRICKLE1)/RE1‐silencing transcription factor (REST) axis. Further mechanistic studies reveal that mGluR1 interacts directly with and stabilizes EGFR in a glutamate‐dependent manner, supporting LC cell survival, colonization, and progression in the brain [482]. In BC, high mGluR1 expression similarly enhances tumor invasion and metastasis; thus, mGluR1 may indicate poor distal metastasis‐free survival [483]. Speyer et al. reported that riluzole, a glutamate release inhibitor, demonstrates therapeutic efficacy in BC [484]. In medulloblastoma, mGluR4 expression is negatively correlated with tumor invasiveness and recurrence [485].

Glutamate can indirectly affect cancer cells via immunomodulatory functions (Figure 4C). Glutamate transporters and receptors, including mGluR1, mGluR5, AMPA, and NMDA, and kainate receptors, are expressed in immune cells [453, 454 ^,^ 486, 487, 488, 489]. NMDA receptors are upregulated following lymphocyte activation [490]. Studies indicate that glutamate‐rich TME promotes tumor development and suppress antitumor immunity. The release of a large amount of glutamate by tumor cells reduces the cytotoxicity of neutrophils. Glutamate‐mediated increases in pSTAT3/RAB10/ARF4 are associated with the immunosuppressive phenotype of neutrophils in the TME [491]. Activation of mGluR1 in MDA‐MB‐231 BC cells promotes the release of immunosuppressive cytokines and chemokines (CXCL1, IL6, IL8), which activate immunosuppressive immune cells, such as neutrophils, accelerating malignant transformation [492, 493]. Interference with mGluR4 enhances antitumor immunity by activating NK, CD4^+^ T, and CD8^+^ T cells. Gene knockout or pharmacological inhibition of mGluR4 stimulates CD8^+^ T cells to produce IFN‐γ through the cAMP/CREB pathway [494].

#### 2.6.2 Glutamate in NDDs

Elevated glutamate exacerbates DA neuron damage (Figure 4D). Patients with PD exhibit higher plasma glutamate, and PD models show approximately doubled striatal extracellular glutamate. The neurotoxin MPTP, used in PD models, is converted to MPP^+^ in astrocytes, activating the NF‐κB/JNK/c‐Jun pathway to downregulate glutamate transporter‐1 (GLT‐1), thereby reducing glutamate uptake and causing excitotoxicity. Interventions such as ceftriaxone treatment, neural precursor cell expressed, developmentally downregulated protein 4‐2 (Nedd4‐2) knockdown, or miR‐543‐3p/342‐3p inhibition mitigate this effect [495, 496, 497, 498, 499]. Receptor dysfunction also contributes to pathogenesis. Group I mGluRs on DA neuron terminals inhibit DA release, and their antagonists reduce nigral degeneration. The NMDA receptor antagonist MK‐801 suppresses glutamate‐induced excitotoxicity; however, excessive inhibition may induce anxiety or impair executive function [500, 501].

Additionally, neuronal death and synaptic glutamate overflow triggers a vicious cycle. Excessive glutamate increases mitochondrial Ca^2+^ influx and ROS production, activating microglia and astrocytes to secrete proinflammatory factors. Overactivated glia subsequently downregulate EAATs and upregulate glutamate receptors, further amplifying glutamatergic excitotoxicity [502, 503].

Physiologically, glutamate is recycled via the glutamate–GABA–glutamine cycle (Figure 4D). Astrocytes take up glutamate and convert it to glutamine, which neurons then reabsorb, regenerate into glutamate, and store via vesicular glutamate transporters 1 and 2 (VGLUT1/2) [504, 505]. In AD models, progressive EAAT2 downregulation reduces glutamate uptake, and Aβ impairs astrocytic glutamate clearance [506, 507, 508, 509, 510, 511]. This deficit leads to chronic synaptic glutamate accumulation, a key driver of neuronal excitotoxicity and synaptic dysfunction in AD.

Aβ further disrupts glutamatergic signaling. Soluble Aβ promotes presynaptic glutamate release and microglial glutamate secretion via soluble amyloid precursor protein (sAPP)/TNF‐α. In vivo, Aβ infusion elevates extracellular glutamate, and impaired clearance near Aβ plaques worsens accumulation [509, 512]. Excessive glutamate accelerates pathology by activating extrasynaptic NMDA receptors, upregulating tau, and inhibiting CREB signaling, while also inducing microglial ROS/proinflammatory factors, forming an Aβ deposition‐glutamate accumulation‐neuronal injury‐impaired Aβ clearance cycle [513, 514].

#### 2.6.3 Glutamate in IBD

Glutamatergic neurons are not widely distributed in the ENS but constitute a specific subgroup marked by the VGLUT2, accounting for only 2%–3% of intestinal neurons. Despite their limited numbers, their regulation of intestinal motility is critically important. VGLUT2 knockout disrupts or accelerates intestinal motility, whereas optogenetic stimulation of these neurons initiates colonic propulsion [515].

Dysregulated glutamate has been implicated in IBD pathogenesis. Patients with IBD and animal models exhibit abnormal mucosal glutamate and VGLUT2 expression, which overactivates enteric neurons, causing diarrhea and spasms, or stimulates macrophages and DCs to release TNF‐α and IL‐1β. In DSS/TNBS‐induced colitis models, VGLUT2 upregulation increases glutamate levels, enhancing neuronal excitability and intestinal barrier permeability [515, 516]. Glutamate also modulates the CAP by activating mGluR5 on intestinal glia to release cytokines and neurotrophins; imbalance between glutamate and ACh disrupts immune homeostasis [516].

In summary, glutamate, a key substance serving as both an energy substrate and a signaling molecule, exhibits abnormal metabolism and signaling pathways mediated by transporters (e.g., EAATs, VGLUT2) and receptors (iGluRs, mGluRs). It plays a central role in tumors, NDDs, and IBD, demonstrating disease‐specific and bidirectional effects. In tumors, abnormal glutamate drives malignant progression. Excessive release by tumor cells or accumulation due to EAAT2 deficiency activates pathways such as PI3K/AKT via mGluR1 and NMDAR to promote proliferation. It also enhances metastasis through mechanisms including mGluR2 downregulation. In NDDs, glutamate excitotoxicity is the core. In PD, GLT‐1 downregulation impairs glutamate clearance, damaging dopaminergic neurons. In AD, Aβ disrupts the glutamate–glutamine cycle, and EAAT2 deficiency causes glutamate accumulation, activating extrasynaptic NMDA receptors and accelerating neuronal damage. In IBD, intestinal glutamate homeostasis imbalance induces pathogenesis. VGLUT2‐positive neurons regulate intestinal motility, and their increased activity or upregulation leads to glutamate accumulation, disrupting motility and triggering proinflammatory cytokine release. Overall, glutamate and its transporter–receptor network constitute a pathological core across multiple diseases. Analyzing the functions of glutamate subtypes and their pathway crosstalk may provide insights for precise therapeutic interventions.

### 2.7 GABA

GABA is a primary inhibitory neurotransmitter in the CNS and mediates more than 40% of inhibitory nerve conduction. It reduces neuronal excitability in the CNS and affects sleep, mood, and cognitive function [13]. Glutamate is the precursor for GABA synthesis, which is catalyzed by glutamate decarboxylases (GAD65 and GAD67 encoded by *GAD1* and *GAD2*) [517]. GAD65 is responsible for the synthesis of GABA for synaptic transmission, whereas GAD67 regulates the production of GABA during metabolic activities [518]. After GABA exerts its function, it is taken up by GABA transporters (GATs) and metabolized by glutaminase and GABA aminotransferase (ABAT) to regenerate glutamate [519]. In the nervous system, GABA plays an important role in the differentiation, proliferation, and migration of neuronal progenitor cells, as well as in neurite elongation and the formation of synapses [448]. GABA deficiency has been linked to cognitive deficits and disorders such as schizophrenia [520]. In addition, GABA is widely distributed in peripheral endocrine organs, including the pituitary gland, pancreas, gastrointestinal tract, and adrenal medulla [521]. GABA is upregulated in some solid tumors, including colon cancer, stomach cancer, ovarian cancer, and BC [522, 523, 524, 525, 526]. GABA acts through GABA receptors of two types: (a) ionotropic GABA_A_ and GABA_C_ receptors, which are ligand‐gated ion channels allowing Cl^−^ flow and forming heteromeric complexes of five subunits; and (b) metabotropic GABA_B_ receptors, which belong to the GPCR family, function as auto‐ or heteroreceptors to limit the release of GABA, glutamate, or other neurotransmitters, and are associated with chemokine and catecholaminergic receptors [527]. GABA_A_ receptors have at least 19 subunits in humans. GABA_A_ receptors are heterodimers consisting of GABA_B1_ and GABA_B2_ subunits. GABA_B1_ has at least 14 subtypes, including GABA‐binding domains, of which GABA_Bla_ and GABA_B1b_ are the most abundant. GABA_B2_ subunits are coupled to the G‐protein complex [484]. GABA receptors are expressed in various tumor tissues and can influence tumor initiation and progression [522, 528, 529].

Building on the above overview, the following sections summarize the role of GABA in two key pathological contexts, including cancer and NDDs, with a focus on regulatory effects and underlying molecular pathways (Figure 4).

#### 2.7.1 GABA in Cancer

The effect of GABA on tumor cell proliferation is cancer‐specific and receptor‐type‐dependent, with the expression of GABA_A_ receptors upregulated in PCa, BC, and PC (Figure 4E) [530, 531, 532]. In contrast, GABA_B_ receptors are downregulated in liver cancer and PC [89, 533]. Generally, activation of the GABA_A_ receptor pathway promotes tumor proliferation, whereas activation of the GABA_B_ receptor pathway inhibits tumor proliferation [529]. In PC, GABA functions in accordance with this principle [534]. Mechanistically, GABA activates the Ca^2+^/MAPK/ERK cascade through the GABRP‐overexpressing GABA_A_ subunit, thereby stimulating PC growth [535]. Conversely, GABA_B_ receptor activation suppresses luciferase activity of p‐CREB and cAMP, reduces isoproterenol‐induced ERK1/2 phosphorylation, and inhibits DNA synthesis [536]. Notably, the π subunit of GABA_A_ receptors can also regulate KCNN4‐mediated Ca^2+^ signaling independently of GABA, thereby promoting PC progression [531]. In BC, GABA, GABA receptors, and GAD are significantly elevated compared with normal breast tissue, and GABA expression correlates with disease stage, suggesting that the GABAergic system contributes to the regulation of BC [525, 537]. In brain metastases of BC, metastatic cells acquire neural‐like features with increased GABA_A_ receptor, GAT, and GABA aminotransferase expression. This enables GABA and succinate accumulation, enhancing NADH biosynthesis and promoting proliferation [538]. Conversely, GABA_B_ receptor activation exerts antiproliferative effects, as confirmed in GABA_B_ agonist experiments [484]. In CRC, GABA levels are elevated, and high GAD1 expression is associated with poorer survival in stage T3/T4 CRC. In a study by Qiu et al., the GABA‐degrading enzyme ABAT is downregulated in CRC compared with normal tissue [539]. These findings suggest that the GABAergic system influences CRC pathophysiology, although the underlying preclinical mechanisms remain unclear. In vitro, GABA decreased proliferation in 5‐FU‐resistant HT29 cells, which resisted the killing effect of 5‐FU, but did not affect parental HT29 or SW480 cells [540], suggesting that tumor responses may depend on cellular context or external conditions [13]. Nevertheless, a study by Song et al. has demonstrated that GABA alone inhibits proliferation of CRC cell lines such as SW620 and SW480 in xenografted nude mouse models [541]. Hence, the effect of GABA on CRC proliferation requires further classification. In GC, activation of the GABA_A_ receptor by muscimol stimulates the MAPK pathway, thereby promoting cell proliferation [535]. In contrast, baclofen, a GABA_B_ receptor agonist, inhibits HCC progression by downregulating intracellular cAMP levels and upregulating p21(WAF1) [89]. Mechanically, GABA‐mediated suppression of GSK‐3β activity enhances β‐catenin signaling, leading to promoted proliferation of tumor cells [542].

In BC, the π subunit of GABA_A_ receptors facilitates TNBC cell invasion via ERK1/2 activation, while the α3 subunit activates AKT signaling to promote cell migration (Figure 4F) [543]. Similarly, GABA and baclofen enhance PC cell invasion by inducing EGFR transactivation [544]. In colon cancer, however, GABA and baclofen suppress SW480 cell migration, likely through GABA_B_ receptor‐mediated inhibition of EMT and Hippo/YAP1 pathways, which reduces intracellular cAMP and modulates PKA activation [11, 545, 546]. The knockdown of GABA_B1_ receptors increases EMT markers in LOVO and RKO CRC cells and promotes metastasis, suggesting an antitumor role for the GABA_B_ subunit. Furthermore, propofol, a GABA_A_ receptor agonist, reduces LOVO cell invasion [88]. Conversely, GABA promotes tumor progression via tumor‐derived exosome miR‐223‐3p, which activates macrophage MAPK signaling, induces M2 polarization of macrophages, and promotes IL17‐secretion, thereby stimulating tumor proliferation and migration. Downregulation of endogenous miR‐223‐3p targets the E3 ligase casitas B‐lineage lymphoma proto‐oncogene B (CBLB), stabilizing c‐Myc and further enhancing CRC proliferation and migration [547]. In HCC, GABA directly binds and stabilizes β‐catenin, activating Wnt/β‐catenin signaling and promoting metastasis. β‐catenin, in turn, upregulates SLC6A12 transcription, facilitating GABA uptake and forming a positive feedback loop [548]. In NSCLC, GABA activates the NF‐κB pathway and astrocytes through the forkhead box protein A2 (FOXA2)/ABAT/GABA axis, contributing to brain metastasis development [549]. In LUAD, GAD1 is overexpressed, and elevated GABA suppresses NF‐κB and STAT3 pathways, inhibiting M1 macrophage polarization while promoting M2 polarization via STAT6 activation. GABA also promotes tumor neovascularization by increasing the expression of fibroblast growth factor 2 (FGF2) in macrophages. GABA_A_ receptor inhibitors exhibit therapeutic efficacy in mouse tumor models, reducing tumor size and body weight [550].

The GABAergic signaling system also plays an important role in antitumor immunity (Figure 4G) [4]. Macrophages, DC, T cells, and B cells express GABA receptors [551, 552, 553, 554]. Regulation of GABA_A_ receptors mediates the polarization of macrophages [555], migration of DC [556], secretion of cytokines [557], cytotoxicity of immune effector cells, and inhibition of T cell proliferation [558]. GABRP overexpression in BC, GC, and other cancers promotes immune evasion by inducing excessive GABA production, thereby recruiting Tregs and M2 macrophages to establish an immunosuppressive TME [559]. In PDAC, elevated GABA receptors interact with KCNN4, augmenting Ca^2+^ influx, activating the KCNN4 pathway, and promoting macrophage infiltration through CXCL5 and CCL20 induction [531]. Additionally, GABA‐mediated activation of GABA receptors inhibits DC‐mediated T cell recruitment [542] and suppresses GSK‐3β activity, thereby enhancing β‐catenin signaling and inhibiting CD8^+^ T cell infiltration [13, 542]. GABA signaling also modulates the function of antigen‐presenting cells (APCs), inhibiting T cell activation and proinflammatory production [560, 561]. In HCC, mevalonate phosphate kinase (PMVK) phosphorylates and stabilizes GAD1, enhancing GABA synthesis. PMVK also recruits acetyl‐CoA acetyltransferase 1 (ACAT1), enabling conversion of GABA into 4‐acetylaminobutyric acid (4‐Ac‐GABA), which is released into the TME. In the TME, 4‐Ac‐GABA activates GABA_A_ receptors on CD8^+^ T cells, inhibiting ACT1 signaling and suppressing CD8^+^ T cell activation, intratumoral infiltration, and antitumor responses [562]. Activated B cells can also synthesize GABA via GAD67‐mediated conversion of glutamate and secrete GABA that binds to GABA_A_ receptors on macrophages and CD8^+^ T cells, triggering reduced intracellular Ca^2+^ and Cl^−^, M2 macrophage polarization, and suppressed [563] CD8^+^ T cell cytotoxicity [563, 564]. Knockout of GAD67 (GAD1) in B cells decreases GABA production and enhances antitumor responses [564]. Benzodiazepines, which enhance GABA_A_ receptor‐mediated anion transport, can depolarize melanoma cells, suppress tumor growth, and increase radiosensitivity and immune checkpoint inhibitor efficacy by promoting CD8^+^ T cell infiltration [565].

#### 2.7.2 GABA in NDDs

GABAergic neuron dysfunction in the basal ganglia is universal in PD [566, 567]. Dopaminergic neuron degeneration correlates with reduced GABAergic neurotransmission, which originates from striatal DA‐GABA corelease. Vesicular monoamine transporter 2 (VMAT2) regulates GABA release, and dopaminergic neurons take up GABA via GAT for cosecretion with DA [568]. Selective D2R depletion in the indirect pathway reduces GABAergic transmission and induces severe motor dysfunction, confirming DA‐GABA crosstalk is essential for motor function [569]. Physiologically, GABA inhibits Ca^2+^ overload and Lewy body deposition, thereby preventing neuronal hyperexcitability [570]. In PD, reduced GAD expression and GABAergic dysfunction cause Ca^2+^ accumulation, which triggers excitotoxicity, oxidative stress, and mitochondrial dysfunction, and promotes aS aggregation and dopaminergic damage [571, 572, 573, 574].

GABA_A_ receptor expression is brain‐region specific (elevated in the cortex and reduced in the thalamus) [575]. GABA modulators demonstrate therapeutic potential: baclofen/clonazepam inhibit glutamatergic transmission, enhance GABA signaling, and protect dopaminergic neurons [576]. The GABA_A_ agonist zolpidem reduces motor dysfunction [577], and the GABA_B_ agonist baclofen alleviates MPTP‐induced PD motor deficits (an effect reversed by the GABA_B_ antagonist CGP35348) [578].

Although historically less studied than glutamatergic signaling, GABAergic dysfunction drives AD pathology via neural circuit imbalance, neuronal injury, and impaired pathological clearance. Early‐stage AD exhibits neuronal hyperexcitability due to weakened GABAergic inhibition, which triggers hippocampal seizures and disrupts synaptic plasticity [579, 580, 581, 582, 583, 584, 585, 586]. In addition, GABA receptor and transporter expression are regionally altered. Cortical and hippocampal GABA uptake are reduced, accompanied by downregulation of GAT1 and GAT3 and BGT1 upregulation [587, 588, 589, 590]. Metabolic dysfunction includes decreased GABA transaminase (GABA‐T) activity and impaired GABA oxidative metabolism in 5 × FAD mice [591, 592]. Aβ is also toxic to GABAergic neurons; their density decreases around Aβ plaques, and Aβ induces hippocampal GABAergic neuron Ca^2+^ efflux and synaptic dysfunction [593, 594, 595]. This reduces GABA levels in the temporal cortex and CSF [596].

Astrocyte–GABA interactions are also critical. Reduced EAAT2 expression impairs glutamate clearance and limits the GABA precursor supply, an effect that can be reversed by ceftriaxone [506, 509, 597]. Meanwhile, reactive astrocytes abnormally release GABA (via GAT3 reverse transport in 5 × FAD and MAO‐B/Best1 in APP/PS1 models), enhancing tonic inhibition and disrupting memory [585, 586]. Microglial GABA signaling is bidirectional; it suppresses inflammation via NF‐κB/p38 downregulation but may activate NLRP3 inflammasomes to promote inflammation [598, 599, 600, 601]. Importantly, GABA regulates microglial Aβ uptake, and GABA_A_ antagonists block γ‐oscillation‐induced Aβ clearance in 5 × FAD mice [601].

In summary, GABA, as the primary inhibitory neurotransmitter in the CNS and a key signaling molecule in the TME, exerts its biological effects through GABA_A_ GABA_B_ receptors, and transporters (e.g., GATs), exhibiting significant disease specificity, receptor subtype dependence, and bidirectional regulatory properties. In cancer, GABA's regulation of malignant progression varies by tumor type and receptor subtype. In terms of proliferation, GABA_A_ receptors primarily exert protumorigenic effects, promoting the progression of PCa, BC, and GC through Ca^2+^/MAPK/ERK and Wnt/β‐catenin pathways. Conversely, GABA_B_ receptors show tumor‐suppressive effects, inhibiting the proliferation of HCC and CRC by downregulating cAMP and blocking the Hippo/YAP1 pathway. In metastasis, GABA_A_ receptors drive the metastasis of BC and NSCLC via the ERK1/2 and AKT pathways, whereas GABA_B_ receptors inhibit CRC invasion by suppressing EMT. In terms of immunity, GABA shapes an immunosuppressive microenvironment by recruiting Tregs, promoting M2‐type macrophage polarization, and inhibiting CD8^+^ T‐cell activation and infiltration. In NDDs, dysfunction of the GABAergic system represents a core pathological mechanism. In PD, degeneration of SbN dopaminergic neurons disrupts DA‐GABA crosstalk, and reduced expression of GAD causes GABA deficiency, leading to Ca^2+^ overload and aS aggregation, which exacerbates neuronal injury. In AD, weakened GABAergic inhibition induces neuronal hyperexcitability, and downregulation of GAT1/GAT3 impairs GABA clearance, while Aβ directly damages GABAergic neurons. Additionally, abnormal GABA release by astrocytes and bidirectional regulation of inflammation and Aβ clearance through microglial GABA signaling collectively exacerbate cognitive impairment. Overall, the dynamic balance of GABA and its receptor–transporter network constitutes a key node in maintaining pathological homeostasis across multiple diseases. Comprehensive analysis of receptor subtype‐specific functions, signaling crosstalk, and disease stage‐dependent effects can provide theoretical support for precise targeted therapy in cancer and NDDs.

### 2.8 Neuropeptides

Neuropeptides are signaling molecules in nervous tissues that regulate nervous system functions. They control pain, sleep, mood, and memory, and also act peripherally as paracrine and endocrine factors, modulating physiological activities such as smooth muscle contraction, fluid homeostasis, and inflammation [602]. Moreover, certain neuropeptides, including SP and NPY, play key roles in cancer progression. SP is synthesized by macrophages, neuronal cells and epithelial cells [603, 604], and regulates neurogenic inflammation, immune responses, and biogenic activities such as movement, sensory perception, neuronal survival and degeneration, and respiration [605]. SP exerts its effects primarily through neurokinin receptors, including neurokinin 1R (NK‐1R), neurokinin 2R (NK‐2R), and neurokinin 3R (NK‐3R), with the highest affinity for NK‐1R [606]. NK‐1R, a GPCR, mediates intracellular signaling by inducing IP3 hydrolysis, calcium influx, and MAPK activation, and influences various physiological and pathophysiological responses, including cell proliferation, migration, and inflammation. It has also been identified in multiple cancer subtypes [92, 607, 608, 609, 610, 611]. NPY is one of the most widely expressed neurotransmitters in the CNS and PNS. It regulates stress responses, circadian rhythm, and cardiovascular function. Research on NPY's effects on aging, metabolic disorders, and cancer remains important [612]. NPY actions are mediated by several NPY receptor subtypes (Y1R–Y6R), with Y1R, Y2R, Y4R, and Y5R extensively characterized. Postsynaptic Y1R directly or indirectly mediates vasoconstriction by enhancing NE activity and stimulates smooth muscle cell proliferation. Both Y1R and Y5R contribute to the formation of atherosclerotic plaques, whereas Y2R promotes endothelial cell progression, migration, and capillary angiogenesis, acting synergistically with Y5R. Additionally, Y2R inhibits presynaptic NE release [613].

**FIGURE 5:**
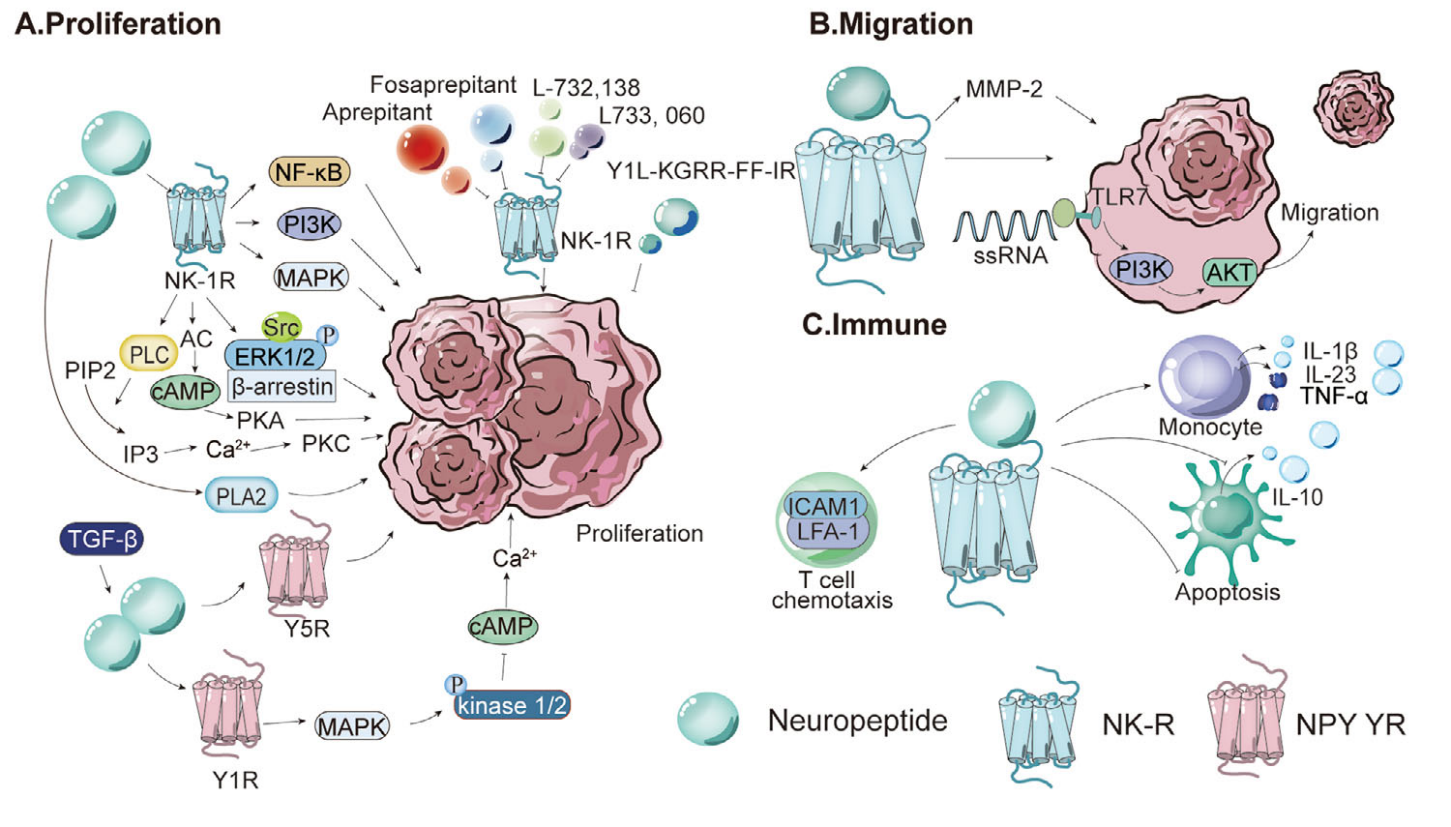
Role and mechanism of neuropeptides in tumorigenesis and development. (A) Certain neuropeptides, such as SP and NPY, play key roles in cancer progression. SP acts as a mitogen in cancer cells expressing NK‐1R, promoting the proliferation of tumor cells. SP activates NK‐1R and induces signaling pathways, including the PI3K, NF‐κB, and MAPK pathways, to promote cancer cell proliferation and survival. NK‐1R inhibitors, such as aprepitant, fosaprepitant, L‐732,138, and L‐733,060, suppress cancer cell proliferation. Similarly, NPY can promote tumor growth through the TGF‐β/NPY/NPY Y5 receptor (Y5R) pathway and the NPY Y1 receptor (Y1R)/MAPK pathway. (B) SP and NPY also promote cancer cell metastasis. The death of a subset of NK‐1R‐highly expressing tumor cells releases ssRNAs, which act on TLR7 in adjacent tumor cells, activating the PI3K/AKT signaling pathway and thereby promoting metastasis. (C) SP and NPY may indirectly regulate tumor progression by modulating immune cells. SP generally stimulates immunity; activation of the SP/NK‐1R system affects immune responses and the function of various inflammatory cells. NK‐1R signaling reduces the apoptosis of DCs and inhibits the secretion of IL‐10, promoting the differentiation of DCs toward a type 1 immune response. Additionally, SP locally acts on memory T cells by inducing IL‐1β, IL‐23, and TNF‐like 1α in monocytes, influencing the proliferation of bone marrow monocytes.

Building on this overview, the following sections summarize the role of neuropeptides in cancer, NDDs, and inflammatory diseases, focusing on their regulatory effects and underlying molecular pathways (Figure 5).

#### 2.8.1 Neuropeptides in Cancer

Both SP and NK‐1R are overexpressed in a variety of cancers such as BC, colon cancer, ovarian cancer, PCa, PC, thyroid cancer, and GBM (Figure 5A). Based on previous research, SP may act as a mitogen in cancer cells expressing NK‐1R, promoting tumor cell proliferation. In BC, SP activates the NK‐1R and induces signaling, including the PI3K, NF‐κB pathway, and MAPK, to promote the proliferation and survival of cancer cells. In human PC samples, NK‐1R mRNA and protein levels are significantly elevated, which is related to poor prognosis. Treatment with SP promotes the propagation of MIA PaCa‐2 and BxPC‐3 cells; conversely, NK‐1R antagonists control PC growth [614]. Additionally, SP induces mitosis in various human tumor cell lines such as astrocytoma, retinoblastoma, glioma, and LC [610].

Mechanistically, SP activates the NK‐1R and induces a scaffold complex assembly, which is composed of internalized receptors, β‐arrestin, Src, and ERK1/2. ERK1/2 translocates to the nucleus, promoting its propagation and inhibiting apoptosis [615, 616]. Three second‐messenger systems are also activated, stimulating phosphatidylinositol conversion through PLC, causing intracellular and extracellular Ca^2+^ mobilization; cAMP accumulation is stimulated by AC, and arachidonic acid is mobilized through PLA2. SP also primes glycogen breakdown to provide energy for tumor cell growth [605, 617]. Pharmacological blockade of NK‐1Rs using specific antagonists (aprepitant, fosaprepitant, L‐732, 138, and L‐733, 060) can have a significant antitumor effect [90, 91, 92, 93, 618, 619]. Similar to SP, NPY can also affect various cancers, such as PCa, BC, and neuroblastoma, and is generally assumed to be a growth‐promoting factor [620]. BC and adrenal, renal, and ovarian cancer cells express Y1R and Y2R [621, 622, 623]. A lysosome‐targeted, enzyme‐responsive precursor (Y1L‐KGRR‐FF‐IR) was developed based on NPY Y1R. Upon entry into lysosomes, it self‐assembles into nanoscale assemblies, which subsequently exert significant disruptive effects on mitochondria and the cytoskeleton, ultimately inducing apoptosis in BC cells [624]. In BC, NPY mediates 4T1 cell proliferation through NPY Y5R [625]. In liver cancer, NPY accelerates the progression through the transforming growth factor‐β (TGF‐β)/NPY/NPY Y5R pathway [626]. Abnormal overexpression of NPY and NPY Y1R in PCa has also been reported, which modulates kinase 1/2 phosphorylation through MAPK/extracellular signaling [627], reduces cAMP accumulation, and increases calcium and potassium channels [621, 628]. Consistent with SP, NPY also regulates cancer cells’ energy metabolism and promotes PCa cell proliferation by NF‐κB [626].

SP and NPY promoted cancer cell metastasis (Figure 5B). Moreover, they can induce angiogenesis in vivo by stimulating endothelial cells [629, 630]. In PC, the in vitro application of SP increased the aggressiveness of BxPC‐3 and MIA PaCa‐2 cells. SP enhances the migration of PC cancer cells and MMP‐2‐mediated nerve infiltration into the dorsal root ganglion (DRG) by acting on NK‐1R. NK‐1R antagonists can reverse these biological effects. In contrast, in a mouse model of PC, the inhibition of SP signaling reduced tumor volume and microvascular formation [614]. In highly metastatic BC, the axon guidance molecule slit guidance ligand 2 (SLIT2) in the tumor vasculature drives increased innervation. Meanwhile, BC cells induce spontaneous calcium activity in sensory neurons and trigger the release of SP. SP acts on the NK‐1R to drive the death of a small subset of NK‐1R‐highly expressing cancer cells. Single‐stranded RNAs (ssRNAs) released by dying cells then act on toll‐like receptor 7 (TLR7) in adjacent tumor cells, activating PI3K/AKT signaling to promote breast tumor growth, invasion, and metastasis, while enhancing lymph node metastatic spread. The NK‐1R antagonist aprepitant can inhibit BC growth and metastasis [631]. For NPY in PCa cells, the number of NPY^+^ nerves increased and the growth of NPY‐specific neurites increased, which was related to the aggressive behavior of the tumor. Simultaneously, inhibition of NPY signaling reduces the migration of cancer cells, promotes apoptosis, and alters energy metabolism [626]. NPY was also detected in PC samples. Y2R is significantly increased in both mouse and human PanIN lesions and PC samples, and the enhanced Y2R‐mediated NPY signaling pathway might regulate PC angiogenesis [632].

SP and NPY can also indirectly regulate tumor progression by affecting immune cells (Figure 5C). In general, SP stimulates the immune system. Activation of the SP/NK‐1R system can affect immune function and alter the responses of various inflammatory cells. NK‐1R is expressed in several immune cell types, such as NK cells [633], DCs [634], T cells, B cells [635], macrophages [636], eosinophils, and mast cells [637]. The activation of NK‐1R signaling can reduce DC apoptosis, and the NK‐1R signaling pathway can inhibit IL‐10 secretion, thereby promoting immune‐stimulated DCs capable of favoring type 1 immunity. NK‐1R activation in lymphocytes affects T cell proliferation, differentiation, and cytokine production [635, 638, 639, 640, 641, 642, 643, 644]. SP affects the proliferation of bone marrow mononuclear cells, acting on memory T cells locally by inducing levels of IL‐1β, IL‐23, and TNF‐like 1α in monocytes [645]. Additionally, SP enhances T‐cell chemotaxis by enhancing T‐cell adhesion to lymphocyte function‐associated antigen‐1 (LFA‐1) and intercellular adhesion molecule 1 (ICAM‐1) in endothelial cells [639, 646]. NPY released by cancer cells can act on receptors expressed on immune cells, alter tumor‐related angiogenesis and inflammation, and promote cell growth [4]. NPY also regulates immune cell transport and alters macrophage function. NE‐treated Myc‐CaP cells promote NPY release, leading to bone marrow cell infiltration, which leads to the elevation of IL‐6 levels in TAM in the PCa TME [647]. There are few studies on neuropeptides that require further exploration.

#### 2.8.2 Neuropeptides in NDDs

Neuropeptides such as SP, NPY, and CGRP exert neuroprotective effects in PD via antiapoptotic, antioxidative, and anti‐inflammatory mechanisms, although certain roles are controversial [648, 649, 650, 651, 652, 653, 654].

SP shows a rise‐then‐fall pattern in 6‐OHDA PD models, whereas patients with PD exhibit reduced SP levels in the SbN and outer paleostriatum [655, 656, 657]. Functionally, low concentrations of SP act on NK‐1R, block Ca^2+^ influx, and suppress caspase‐3 activation to protect MPP^+^‐treated MES23.5 cells. By contrast, exogenous SP exacerbates dopaminergic cell death and motor deficits. SP also promotes microglial recruitment via the NK‐1R/PKC/nicotinamide adenine dinucleotide phosphate hydrogen (NADPH) oxidase pathway [649, 656, 658, 659, 660, 661].

NPY levels are reduced in the adrenal medulla and CSF of patients with PD [662]. Its neuroprotective effects are Y2R‐dependent, and Y2R knockout abolishes protection in 6‐OHDA models. NPY activates Akt/MAPK signaling to support dopaminergic neurons. It suppresses glial activation, NO/IL‐1β production, and alleviates ER stress via PI3K, X‐box binding protein 1 spliced (XBP1s), and binding immunoglobulin protein (BiP) [663, 664, 665, 666].

Similarly, in AD, SP, NPY, and CGRP modulate neuroinflammation, synaptic plasticity, and vascular function.

Patients with AD exhibit reduced CSF and hippocampal SP levels, which correlate with neurofibrillary tangles and cognitive decline. SP activates microglia via NK‐1R/PKC/NADPH oxidase, exacerbating inflammation, but mitigates synaptic toxicity at low concentrations, whereas high concentrations promote tau hyperphosphorylation [649, 656, 660, 661]. NK‐1R antagonists such as NAT and ranipirtan inhibit excessive microglial activation, reduce Aβ‐induced neuronal damage, and alleviate levodopa‐related dyskinesia, suggesting anti‐inflammatory and neuroprotective potential [656, 667].

NPY is reduced in the CSF, correlating with synaptic loss, whereas NPY mRNA in the nucleus accumbens and caudate is elevated as a compensatory response. It maintains synaptic balance via Y2R, reverses Aβ‐induced long‐term potentiation (LTP) suppression, and suppresses inflammation and ER stress [663, 665, 666, 668, 669, 670].

CGRP levels are reduced in the CSF (CGRP‐α), correlating with cognitive decline. It improves cerebral blood flow, promotes neurogenesis and synaptic plasticity via cAMP signaling, and modulates microglial inflammation. Its antimicrobial properties regulate gut microbiota, potentially influencing AD pathology via the gut–brain axis [671, 672, 673, 674, 675, 676, 677, 678].

#### 2.8.3 Neuropeptides in Inflammatory Diseases

The airway possesses a robust neuropeptide network, which is critical for normal airway physiology and is closely associated with airway diseases such as asthma [679].

SP is widely expressed in neurons, glial cells, immune cells, and some progenitor/stem cells [646]. Its receptors are distributed in the parasympathetic nervous system, airway smooth muscle, and airway epithelium, providing a structural basis for its diverse airway functions [680, 681]. In asthma, serum SP levels are elevated and correlate with disease severity, and SP induces allergic airway inflammation in mice [682]. Specifically, SP released from pulmonary nociceptors expressing FcεR1γ activates IgE–allergen immune complexes via the same receptor, promoting Th2 cell polarization and infiltration [683].

CGRP is widely distributed in nerve fibers innervating multiple organs and pulmonary neuroendocrine cells. It regulates airway function by modulating bronchial smooth muscle tone and airway mucus secretion [684, 685] and may contribute to airway constriction in asthma [686]. Its role in asthma is complex. CGRP levels in bronchoalveolar lavage fluid (BALF) increase during allergen‐induced late‐phase reactions in patients with asthma [687, 688], yet it also exhibits anti‐inflammatory effects, including inhibiting DC maturation and reducing eosinophil counts [684].

Vasoactive intestinal peptide (VIP) is one of the most abundant lung neuropeptides and exerts multifaceted airway effects, including mediating bronchial smooth muscle relaxation and vasodilation [689]. Plasma VIP levels change significantly during asthma exacerbations [690]. VIP also has anti‐inflammatory properties: it inhibits eosinophil migration while inducing bronchodilation, making it a potential therapeutic target [684, 691]. In terms of neuroimmune interactions, IL‐5 from ILC2 and CD4^+^ T cells directly stimulates VIP secretion from pulmonary nociceptors, and VIP in turn activates ILC2 and CD4^+^ T cells, forming a positive feedback loop that amplifies Type 2 inflammation in OVA‐ and HDM‐induced asthma mouse models [692].

Similarly, in the gut, neuroimmune crosstalk mediated by neuropeptides drives IBD [693].

SP is expressed in the enteric plexus and immune cells, acting via NK‐1R [694, 695, 696, 697, 698, 699, 700, 701]. In DSS and TNBS models, SP elevates proinflammatory cytokines (IL‐1β, IL‐6) via MAPK/NF‐κB [702, 703, 704, 705, 706, 707, 708, 709], while also promoting epithelial repair via EGFR deactivation [710]. In patients with IBD, intestinal SP levels are inconsistent [695, 711, 712, 713, 714, 715, 716, 717], but NK‐1R is consistently upregulated [718].

CGRP, originating from DRG (α‐subunit) and ENS neurons (β‐subunit) [719], primarily exerts anti‐inflammatory effects in IBD. It inhibits macrophage and T cell activation, promotes TGF‐β production to alleviate colitis, and suppresses ILC2 activity [720, 721, 722, 723, 724, 725]. In DSS colitis models, CGRP prevents inflammation by inducing T cell apoptosis and macrophage‐derived TGF‐β. TGF‐β‐deficient mice exhibit elevated pro‐inflammatory cytokines, including IFN‐γ, IL‐1β, and TNF‐α [724].

## 3 Potential Therapeutic Targets

As key signaling molecules mediating interactions between the nervous, immune, and metabolic systems, neurotransmitters have become central therapeutic targets for diseases such as PD, AD, MG, asthma, and various cancers. While the main targets and clinical maturity of neurotransmitter‐based treatments vary across these conditions, their development has shifted significantly: from the early, simple approach of supplementing a single neurotransmitter to complex multifaceted strategies that coordinate regulation across multiple systems. Notably, this change includes not just combination drug therapies, such as DA precursors and degradation inhibitors for PD, but also integrated treatments that combine pharmacology with novel technologies. For example, cell replacement therapy for PD aims to rebuild damaged dopaminergic neural circuits. This shift reflects a deeper and more comprehensive understanding of neurotransmitter regulatory networks and their influence on disease development. Against this backdrop, the following sections systematically summarize the current clinical applications of neurotransmitter‐targeting strategies (Table 2). We also provide targeted perspectives to inform the clinical translation and optimization of these strategies.

**TABLE 2:** Clinical therapy of targeting neurotransmitter.

Clinical trials.gov identifier	Disease	Drug	Phase	Targets and mechanism	Effect
NCT00030979	PD	Donepezil	Phase 4	Inhibiting acetylcholinesterase	No signs of symptom deterioration after treatment, well tolerated, and mild adverse reactions
NCT03115827	PD	Droxidopa	Phase 4	Adding NE	Active, not recruiting
NCT00086190	PD	Paroxetine and venlafaxine	Phase 3	Inhibiting NE and serotonin reuptake	Improving depression, safe and well‐tolerated
NCT00914095	PD	Methylphenidate	Phase 4	Inhibiting NE and DA reuptake	Improving motor disorders
NCT01504178	PD	Duloxetine	Phase 3	Inhibiting NE and serotonin reuptake	Improving gait and motor symptoms in the absence of levodopa, and increasing the response intensity to levodopa
NCT06236230	PD	Levodopa, carbidopa, and entacapone	Phase 4	Levodopa, carbidopa: adding DA. Entacapone: inhibiting COMT	Recruiting
NCT00402233	PD	Pramipexole	Phase 4	Activating DAR	Recruiting
NCT01782222	PD	Rotigotine	Phase 4	Activating DAR	Improving the symptoms of PD within 4 weeks
NCT06167681	PD	NouvNeu001	Phase 1, Phase 2	Adding DA	Active, not recruiting
NCT00038116	PD	Embryonic dopamine cell implant surgery	Phase 3	Adding DA	Recruiting
NCT06583291	PD	Allogeneic dopaminergic neural precursor cell	Early Phase 1	Adding DA	Improving motor symptoms in young people
NCT06978920	PD	Allogeneic dopaminergic neural precursor cell (NCR201)	Phase 1	Adding DA	Not yet recruiting
NCT07028632	PD	NouvNeu001 (human dopaminergic progenitor cells injection)	Phase 2	Adding DA	Recruiting
NCT00643045	PD	Safinamide	Phase 3	Inhibiting MAO‐B	Good tolerance as an adjuvant treatment
NCT01382342	PD	Rasagiline	Phase 4	Inhibiting MAO‐B	Not improving the apathy associated with PD, but improving motor and activities
NCT06785298	PD	Fexofenadine	Phase 2, Phase 3	Inhibiting H1R	Maintaining the improvement and stability of symptoms
NCT04497168	PD	Citalopram	Phase 2	Inhibiting serotonin reuptake	Improving the performance of motor symptoms, but no significant changes in any neuropsychological measurements
NCT06785298	PD	Fexofenadine	Phase 2, Phase 3	Inhibiting H1R	Recruiting
NCT01767129	PD	AVP‐923	Phase 2	Inhibiting NMDA receptor	Improving lower axial movement symptoms and movement disorder scores, but not improving gait
NCT01108029	PD	Memantine	Phase 4	Inhibiting NMDA receptor	Improving the Parkinson's Disease Rating Scale score
NCT00001933	AD	Nefiracetam	Phase 2	Enhancing the activity of nAChR by interacting with the PKC pathway and accelerates the metabolism and release of ACh	Improving learning disabilities and memory in rats and patients
NCT05413655	AD	EX039	Phase 2	Acetylcholinesterase inhibitor supplement	Recruiting
NCT01073228	AD	EVP‐6124	Phase 2	Activating alpha‐7 nAChR	Completed
NCT06702124	AD	Rotigotine and rivastigmine	Phase 3	Rotigotine: activating DAR. Rivastigmine: inhibiting acetylcholinesterase	Not yet recruiting
NCT06995573	AD	NSC001	Phase 1, Phase 2	Activating M1R	Not yet recruiting
NCT01009255	AD	GSK239512	Phase 2	Inhibiting H3R	Recruiting
NCT00322153	AD	Memantine	Phase 3	Inhibiting NMDA receptor	Recruiting
NCT03165435	MG	CV‐MG01	Phase 2, Phase 3	AChR mimicry peptide	Withdrawn
NCT00044824 NCT00044811 NCT00045955	Asthma	Fexofenadine	Phase 3	Inhibiting H1R	Improving episodic memory
NCT01847001	BC	Propranolol	Phase 2	Inhibiting β‐AR	The compliance and target measurement of propranolol were obtained
NCT03919461	CRC	Propranolol and etodolac	Phase 2	Propranolol: inhibiting β‐AR. Etodolac: inhibiting COX‐2	Recruiting
NCT03838029	PC	Propranolol and etodolac	Phase 2	Propranolol: inhibiting β‐AR. Etodolac: inhibiting COX‐2	Recruiting
NCT02944201	PDAC	Carvedilol	Phase 2	Inhibiting β‐AR	Recruiting
NCT06225011	CRC	Fluoxetine	Phase 1	Inhibiting serotonin reuptake	Active, not recruiting
NCT05664464	GBM	Gabapentin, sulfasalazine, and memantine	Phase 1, Phase 2	Gabapentin: inhibiting glutamate synthesis enzyme. Sulfasalazine: inhibiting glutamate secretion by blocking the cystine‐glutamate exchanger system Xc Memantine: inhibiting NMDA receptor	Improving motor disorders
NCT04732065	Recurrent Diffuse Midline Gliomas	ONC206	Phase 1	Inhibiting D2R	Recruiting
NCT02288962	Nonfunctioning Pituitary Adenomas	Cabergoline	Phase 3	Activating DA	Active, not recruiting

*Data source*: ClinicalTrials.gov website (https://clinicaltrials.gov/).

For PD, the core pathological feature is DA system imbalance caused by the degeneration of SbN dopaminergic neurons. Enhancing DA signaling is currently the most mature therapeutic strategy. The DAR agonist rotigotine (NCT01782222, Phase IV) directly activates DARs. Clinical trials demonstrate that it significantly improves overall symptoms in patients with PD within 4 weeks and is well‐tolerated long‐term. MAO‐B inhibitors sustain synaptic DA levels by inhibiting its degradation: rasagiline (NCT01382342, Phase IV) effectively improves motor function but has little effect on PD‐associated apathy and other nonmotor symptoms, while safinamide (NCT00643045, Phase III) exhibits excellent safety as an adjunct therapy and is well‐suited for combination with other anti‐PD medications. However, all current interventions share a critical limitation: none can halt or reverse the degeneration of SbN dopaminergic neurons. Clinical management thus remains focused on controlling motor symptoms and addressing nonmotor symptoms. In this context, the triple combination of levodopa, carbidopa (a DA precursor supplement), and entacapone (a COMT inhibitor that reduces DA breakdown; NCT06236230, Phase IV, recruiting) remains the first‐line choice for mid‐to‐late‐stage patients with PD. Its ability to rapidly boost brain DA levels and provide sustained relief from motor symptoms underpins this status. However, DA replacement therapy is limited by the blood–brain barrier (BBB). To address this issue, studies have synthesized two solid lipid nanoparticle (SLN) formulations, namely, DA‐co‐GSE‐SLNs and GSE‐ads‐DA‐SLNs. Studies using differentiated SH‐SY5Y cells have found that these formulations exhibit good biocompatibility, protect cells from rotenone‐induced toxicity and oxidative stress, and regulate the levels of aS. This demonstrates the potential of SLNs as a delivery system to solve PD treatment challenges and enhance drug and antioxidant delivery across the BBB [726]. Meanwhile, to overcome this limitation of merely controlling symptoms, researchers are actively exploring strategies that address the root cause, with cell replacement therapy at the forefront. Embryonic DA cell transplantation (NCT00038116, Phase III, recruiting) aims to rebuild damaged neural circuits by implanting functional dopaminergic neurons. Allogeneic dopaminergic neural progenitor cells (NCT06583291, early Phase I) have shown preliminary motor symptom improvement in young patients with PD. Human DA progenitor cell injections (NouvNeu001, NCT07028632, Phase II, recruiting) further enhance the stability and safety of cell sources, holds potential for the “root‐cause” neural function repair in advanced patients with PD.

For AD, the intervention framework centers on cholinergic system decline, with ACh signaling enhancement strategies being the most mature. Nefuraxetane (NCT00001933, Phase II) enhances nAChR activity by synergizing with the PKC pathway and accelerating ACh release, with approximately 50% of patients in the high‐dose group showing cognitive improvement. The α7 nAChR‐specific agonist EVP‐6124 (NCT01073228, Phase II completed) enables precise receptor subtype modulation, suitable for mild AD. Additionally, the NMDA receptor antagonist memantine (NCT00322153, Phase III recruiting) delays cognitive decline in moderate‐to‐severe patients by inhibiting excessive glutamate activation and is often used synergistically with cholinesterase inhibitors. However, existing therapies cannot reverse core pathologies, such as Aβ deposition and tau phosphorylation, with clinical approaches primarily focused on symptom relief and progression delay. Rotigotine (DA agonist) combined with galantamine (cholinesterase inhibitor; NCT06702124, Phase III not recruiting) explores multineurotransmitter synergy. EX039 (NCT05413655, Phase II recruiting) optimizes AChE inhibitor delivery efficiency. H3R antagonist GSK239512 (NCT01009255, Phase II recruiting) and M1R agonist NSC001 (NCT06995573, Phase I/II not recruiting) expand noncholinergic targets to overcome therapeutic limitations.

For MG, research focuses on correcting autoimmune disorders of the AChR, with the core approach being the induction of immune tolerance. The peptide vaccine CV‐MG01 (NCT03165435, Phase II/III trial withdrawn) triggered tolerance via AChR‐mimetic peptides. Initial validation demonstrated immunogenicity and safety, but the trial was terminated due to failure to meet efficacy endpoints. Currently, no mature neurotransmitter‐targeted therapies exist in clinical practice. The failure of CV‐MG01 highlights issues of insufficient target specificity and limited immunomodulatory efficacy. Future efforts should focus on designing more specific AChR epitope peptides or combining them with PD‐1/PD‐L1 immune checkpoint inhibitors to enhance tolerance induction, aiming to restore NMJ signaling while maintaining immune balance.

For asthma, neurotransmitter‐based interventions primarily target the histaminergic and cholinergic systems, with H1R antagonism as the most established approach. Fexofenadine (NCT00044824, NCT00044811, NCT00045955; all Phase III) selectively blocks peripheral H1R, reducing airway smooth muscle contraction and inflammatory cell infiltration while also improving patients’ episodic memory. It exhibits favorable safety profiles in patients with mild‐to‐moderate asthma. However, no curative neurotransmitter‐targeted therapies exist. A key gap is inadequate regulation of neuropeptides (e.g., SP) and peripheral DA, which drive mucus hypersecretion and exacerbate neurogenic inflammation. Future research should focus on exploiting the synergistic effects of SP/NK‐1R antagonists and histamine inhibitors, as well as developing interventions that modulate neurotransmitter release from airway nerve terminals.

For cancer, neurotransmitter targeting in oncology has emerged as a pivotal interdisciplinary focus, with preliminary frameworks established around the adrenergic, dopaminergic, glutamatergic, and serotonergic systems. However, notable disconnects remain between mechanistic insights and clinical translation. β‐AR inhibition is the most widely applied strategy. In BC, propranolol (NCT01847001, Phase II) blocks sympathetic signaling to reduce metastatic potential. In CRC and PC, the combination of propranolol and etodolac (NCT03919461, and NCT03838029, both Phase II, recruiting) uses a neuroinhibition‐anti‐inflammation approach to counteract immunosuppression. The DA system exhibits bidirectional regulation. In nonfunctioning pituitary adenomas, cabergoline (NCT02288962, Phase III, active) activates D2R to inhibit proliferation, whereas in recurrent diffuse midline gliomas, ONC206 (NCT04732065, Phase I, recruiting) inhibits D2R to induce apoptosis. This divergence stems from tumor differentiation status and crosstalk between receptor downstream pathways.

Overall, three core challenges exist in the current clinical application of neurotransmitter‐targeted therapies. First, there is a lack of curative solutions and difficulty reversing pathological processes. Apart from the core neurotransmitter disorders in PD and AD, which alleviate symptoms, there is a lack of intervention targeting the etiology in the induction of immune tolerance in MG, neurogenic inflammation in asthma, and microenvironment remodeling in tumors. For instance, AD cannot clear Aβ deposits, and tumors cannot block the core mechanisms of neural invasion. Consequently, treatment remains confined to “symptom management” rather than achieving a cure. Second, interventions remain limited to single targets with insufficient multisystem coordination. Most therapeutic approaches focus narrowly on individual neurotransmitters or receptors, overlooking complex interactions within neurotransmitter networks, such as DA‐ACh imbalance in PD. Third, drug delivery technologies suffer from poor targeting, leading to insufficient drug concentrations at intended sites. Physiological barriers, such as the BBB in PD, AD, and GBM, and the complex TME in various cancers, severely hinder drug penetration. Existing delivery systems rely predominantly on passive diffusion and lack active targeting carriers tailored to disease‐specific pathological characteristics.

To address these challenges, future breakthroughs should focus on four key directions. First, deepen mechanistic research by exploring the dynamic interaction networks between neurotransmitters, immune cells, and stromal cells to provide theoretical foundations for multidimensional interventions. Second, advance precision and personalized therapies by stratifying patients based on biomarker detection (e.g., receptor expression levels, genetic typing) and developing tailored treatment plans for populations with high expression of specific targets. For instance, in patients with tumors exhibiting high β‐AR expression, prioritize combination therapy with propranolol and immunotherapy to enhance treatment specificity and efficacy. Third, strengthen combination therapy strategies by actively exploring synergistic multidrug regimens. Combining drugs with distinct mechanisms of action achieves the therapeutic goal of enhancing efficacy while reducing toxicity. Finally, develop targeted drug delivery systems tailored to pathological subtypes: design active targeted delivery vehicles engineered for the physiological barrier characteristics of specific conditions (e.g., BBB, TME) to improve drug utilization. Promising progress already exists here: the neurotransmitter‐mimicking nanovesicle PMVS‐P, loaded with polydopamine and GSC‐targeting salinomycin (SAL), can specifically target D2R‐high GSCs and thus effectively inhibit GBM recurrence [727]. This study validates the feasibility of pathology‐adapted targeted delivery systems, highlighting their potential to overcome barriers in clinical treatment.

## 4 Conclusion and Prospects

In recent years, research at the intersection of neurotransmitters and the immune system has emerged as a key focus. The traditional view held that these two systems functioned independently, but contemporary understanding frames them as forming a neurotransmitter–immune axis. The core significance of this axis extends beyond basic signal transmission. Its key mechanism involves mediating signals via specific receptors on immune cell surfaces and coordinating immune responses through crosstalk between the nervous and immune systems. This mechanism profoundly influences the pathological progression of diverse diseases, including cancer, neurodegenerative disorders (PD, AD), and inflammatory conditions (IBD, asthma). It plays a dual role. On one hand, it disrupts immune homeostasis and triggers pathological processes; for instance, NE induces M2 macrophage polarization via β2‐AR in cancer, while cholinergic deficits impair microglial clearance of Aβ in AD. On the other hand, it serves as a critical therapeutic target, owing to its role as a central hub for neuroimmune interactions.

Neurotransmitters' immunological effects depend on the immune cell receptor subtypes they bind to, the specific immune microenvironment (e.g., tumor immunosuppressive or intestinal inflammatory microenvironments), and the disease's immune stage (e.g., early or chronic inflammation, tumor immune escape, or activation). For example, adrenergic stimulation promotes growth in colon cancer, ovarian cancer, and PC but inhibits melanoma and neuroblastoma [169]. In early‐stage AD mouse models, α7‐nAChRs exert neuroprotective effects by maintaining the septohippocampal cholinergic phenotype [7]. However, another study found that α7‐nAChR knockout mice display neuroprotective effects alongside improved learning and memory [8].

This “single neurotransmitter, dual immunoregulatory effects” trait forms a tight “neuroimmune crosstalk loop” between neurotransmitters and both innate immunity and adaptive immunity. In asthma, allergen stimulation triggers VIP release from sensory neurons. IL‐5 (from ILC2 and CD4^+^ T cells) directly stimulates VIP secretion from pulmonary nociceptors, forming a synergistic pathological chain [692].

Building on the above interaction mechanisms, the core challenge in current neurotransmitter–immune axis targeted therapies lies in overcoming the limitations of “nonspecific interactive regulation.” First, nonselective neurotransmitter drugs, such as β‐AR blockers, act indiscriminately on homologous receptors in both immune and nonimmune cells, leading to immune‐related off‐target effects. For instance, while inhibiting β2‐AR can be used to stabilize the progression of PDAC, it may induce bronchospasm in patients with asthma by acting on β2‐AR in airway smooth muscle cells.

Second, the disease‐specific immune heterogeneity (e.g., varying immune cell infiltration in different cancer types or AD subtypes) results in markedly variable therapeutic responses to the same neurotransmitter target across patients. Third, the “overlapping interactive regulation” of immune responses by multineurotransmitter networks, such as the joint modulation of microglial activation by DA, ACh, and GABA in PD, renders single‐target interventions ineffective at resolving immune dysregulation.

To address these issues, future breakthroughs must focus on precise targeting of “neuroimmune interaction nodes” and adopt a “precision and synergy” dual strategy. On one hand, designing drugs that selectively target receptor subtypes on specific immune cells enables the precision modulation of immune cells without disturbing nonimmune tissues. On the other hand, utilizing combination therapies of neurotransmitter modulators and immunomodulators, such as β‐AR inhibitors combined with PD‐1 antibodies, enhances antitumor immunity. Concurrently, PET receptor imaging can detect neurotransmitter receptor expression on immune cells, thereby aiding in tailoring personalized treatment plans for patients.

In conclusion, research on the neurotransmitter system not only provides molecular targets for the development of innovative therapies but also redefines the understanding of complex diseases as the result of the dysregulation of multiple system networks rather than the abnormality of a single pathway.

With advances in subtype‐selective drug development, the integration of interdisciplinary technologies, and the refinement of biomarker systems, neurotransmitter‐targeted therapies are poised to advance from merely “alleviating immune‐related symptoms” toward “restoring immune homeostasis and blocking disease progression.” This transition stands to drive revolutionary breakthroughs in the treatment of intractable diseases such as cancer and neurodegenerative disorders.

## Author Contributions

Gege Li, Fangfang Li, Siyu Guo, and Yang Tang contributed in manuscript writing. Yihan Yao, Yuan Fang, Yu Jiang, Dang Wu, Bicheng Zhang, and Jing Wang contributed in review discussion and language editing. Ting Zhang, Zengfeng Xin, Jianxia Cheng, and Zhihui Huang participated in the design and review of the manuscript. Gege Li, Fangfang Li, Siyu Guo, and Yang Tang contributed equally to this work. All the authors have read and approved the final manuscript.

## Funding

This work was supported by the grant from the Key Project of Natural Science Foundation of Zhejiang Province (LZ25H160003, Ting Zhang), Beijing CSCO Clinical Oncology Research Foundation (Y‐2020Sciclone/ms‐0099, Ting Zhang and Y‐2019 Sciclone‐019, Ting Zhang), and Shanghai Sixth People's Hospital, Hospital Management Research Center, Hospital Management research project (Lyg12023017, Jing Wang).

## Ethics Statement

The authors have nothing to report.

## Conflicts of Interest

The authors declare no conflicts of interest.

## Data Availability

The authors have nothing to report.
